# Association of Dietary Intake and Biomarker of α-Linolenic Acid With Incident Colorectal Cancer: A Dose-Response Meta-Analysis of Prospective Cohort Studies

**DOI:** 10.3389/fnut.2022.948604

**Published:** 2022-07-07

**Authors:** Ze-Bin Dai, Xiao-Li Ren, Yi-Lang Xue, Ya Tian, Bing-Bing He, Chang-Long Xu, Bo Yang

**Affiliations:** ^1^The Second Affiliated Hospital and Yuying Children's Hospital of Wenzhou Medical University, Wenzhou, China; ^2^Institute of Lipids Medicine, Wenzhou Medical University, Wenzhou, China; ^3^Department of Preventive Medicine, School of Public Health and Management, Wenzhou Medical University, Wenzhou, China

**Keywords:** omega-3 fatty acids, colorectal cancer, meta-analysis, biomarker, linolenic acid

## Abstract

**Background and Objective:**

There is keen interest in better understanding the impacts of alpha-linolenic acid (ALA), a plant-derived n-3 fatty acid, in ameliorating the development of cancer; however, results of several prospective cohorts present an inconsistent association between ALA intake and the incident colorectal cancer (CRC). We aimed to investigate the summary association of dietary intake and biomarkers of ALA with CRC risk based on the prospective cohorts.

**Methods:**

Pertinent prospective cohorts were identified in Cochrane Library, PubMed, and EMBASE from inception to February 2022. Study-specific risk ratios (RRs) with 95% confidence intervals (CIs) for comparing the top with the bottom quartiles of ALA levels were combined using a random-effects model. Nonlinear dose-response relationships of ALA levels in diet and blood with CRC risk were assessed using the restricted cubic spline models, respectively.

**Results:**

Over the duration of follow-up with a median of 9.3 years ranging from 1 to 28 years, 12,239 CRC cases occurred among 861,725 participants from 15 cohorts (11 studies on diet and 5 studies on biomarkers including 4 on blood and 1 on adipose tissue). The summary RR was 1.03 (95% CI: 0.97, 1.10; I^2^: 0.00%) for dietary intake and 0.83 (95% CI: 0.69, 0.99; I^2^: 0.00%) for biomarker. Each 0.1% increase in the levels of ALA in blood was associated with a 10% reduction in risk of CRC (summary RR: 0.90, 95% CI: 0.80, 0.99; I^2^: 38.60%), whereas no significant dose-response association was found between dietary intake of ALA and the incident CRC (p for non-linearity = 0.18; p for linearity = 0.24).

**Conclusions:**

Blood levels of ALA were inversely and linearly associated with the risk of CRC, which suggested that increased intake of ALA to improve circulating levels was beneficial for CRC prevention.

## Introduction

Colorectal cancer (CRC) is the second most common cancer diagnosed in women and the third most in men, and currently ranks as the fourth most deadly cancer worldwide with nearly 900,000 deaths annually (1). The incidence of colorectal cancer worldwide was predicted to be 2.5 million new cases in 2035 (2). As a result, the primary prevention of CRC has always been an important public health priority.

Dietary factors have been shown to play an important role in the prevention of CRC (3). At cellular and animal model levels, dietary n-3 polyunsaturated fatty acids (PUFAs) were proved to be implicated in the several biological mechanisms underlying the antineoplastic effects of alpha-linolenic acid (ALA, 18:3n-3), including suppression of nuclear factor-κB (NF-κB), activation of AMPK/SIRT1, modulation of cyclooxygenase activity, and upregulation of the novel anti-inflammatory lipid mediators identified recently such as protectins, maresins, and resolvins (3, 4).

Alpha-linolenic acid, as a plant-based member of n-3 PUFAs, can be derived from vegetable oils (5). Population-based epidemiological studies have reported the protective effect of ALA on obesity-related diseases such as diabetes and cardiovascular disease (6–8). Nevertheless, the associations with CRC risk were found to be inconsistent in several prior cohorts using food ALA as interest exposure, and two previous meta-analyses reported a null association estimation (9, 10). Given the possibility of a measurement error or report bias in most of the observational cohorts using dietary questionnaires to estimate ALA intake, it was difficult to accurately assess the real intake of individual fatty acids (11). Moreover, there may be a disturbance of gut microbiota in the individuals vulnerable to CRC, which might have resulted in an overestimation of the exact level of ALA *in vivo* (12, 13).

In contrast to dietary questionnaires, biomarker measurements provide objective assessments of ALA exposure in diet, which reflect both on the biologically relevant process and dietary consumption, as well as are free of memory errors, recall bias, or inaccuracies in food databases (14). One prior meta-analysis included three cohorts only to conclude a null association with blood levels of ALA (15), which may have been influenced by the limited number of eligible studies. So far, the relationships between ALA biomarkers and CRC risk remain unclear, as various prospective cohorts reported inconsistent results (16–18). One study found that the levels of ALA in adipose tissue (AT) had an inverse association with the incident CRC (16), whereas the other studies showed that circulating ALA was inversely associated with colon cancer but not rectum cancer (17) and had a null association with CRC risk (18).

To further address the role of the plant-based n-3 fatty acid in preventing the development of CRC, we conducted a meta-analysis to summarize the updated evidence on the relationship between ALA intake and the incident CRC. The novelty of the present study was to quantitatively evaluate a dose-response association of ALA levels in the diet and human biospecimens (blood and AT) with CRC risk using the available data from the more comprehensive perspective studies.

## Materials and Methods

### Literature Search

We identified 20,195 potential studies from PubMed, EMBASE, and Cochrane Library databases up through Feb 2022, and the search strategy we have predefined was listed in the literature searching section of Supplementary Materials. We also searched for the published meta-analyses from the above-mentioned databases and checked their reference lists to identify the relevant publications that might have been missed. The present study was conducted and reported following the Meta-analysis Of Observational Studies in Epidemiology (MOOSE) guidelines (Supplementary Table 2) (19).

### Eligibility Criteria

To assess the association of ALA in diet and human tissues and the risk of CRC, the inclusion criteria were: (1) Participants: Adults of any age across different countries; (2) Exposure: levels of ALA intake estimated by dietary records, food frequency questionnaires, or quantitative determining the compositions or concentrations of ALA in circulating blood and adipose tissue (AT); (3) Outcomes: Evaluating the incident CRC as an endpoint, presented as multivariate-adjusted risk ratio (RR) or hazard ration (HR) with 95% confidence interval (CI); (4) Study design: Prospective cohort study, nested case-control study, and case-cohort study.

### Data Extraction

The following data were extracted by the two independent reviewers from each original study using a standardized extraction form: first author, publication year, study design (prospective cohort/nested case-control/case cohort), study location (America/Europe/Asia), cohort name, sample size (number of cases/participants), baseline age (median value, year), gender, duration of follow-up (median value, year), cancer location (colon/rectum), exposure measurements, types of interest exposure (diet or biomarker), multivariate-adjusted RRs (HRs) with 95% CI for all category levels of ALA in diet or human tissues, and the potential confounders adjusted. The study quality of each included study was evaluated by using the 9-stars Newcastle-Ottawa Scale (NOS) (20) (Supplementary Table 3).

### Data Synthesis

If an original study provided HRs with 95% CIs for the incident CRC, the HR value was assumed to approximate the RR value. All the included studies provided RR (HR) for ALA intake (diet or biomarker) based on various categories (e.g., tertiles, quartiles, or quintiles) or per SD difference in exposure. To achieve a consistent approach to the present meta-analysis, the RRs (HRs) were first transformed to involve comparisons between the top and the bottom quartiles of baseline diet or biomarker of ALA using methods described previously (21, 22). In brief, log risk estimates were transformed with the comparison between the top and bottom quartiles being equivalent to 2.54 times the logRRs for per 1-SD increase. These scaling methods assume that the exposure is normally distributed and the association with the risk of CRC is log-linear. The conversion factor of 2.54 is the difference in the medians of the top and bottom quartiles of the standard normal distribution; other conversions were used for differences in medians of extreme tertiles (2.18) or quintiles (2.80). The standard errors (SEs) of log RRs were calculated using reported data on precision and were similarly standardized.

### Statistical Analysis

Multivariate-adjusted RRs (HRs) comparing the top with the bottom quartiles of ALA intake (diet and biomarker) in each study were first transformed to their logarithm (logRRs), and their corresponding 95% CIs were used to calculate the standard errors (selogRRs). Summary RRs (SRRs) with 95% CIs as the overall risk estimate for the top vs. bottom quartiles of ALA intake was calculated using a random-effects model described by DerSimonian and Laird (23), which considers both within-study and between-study variability. Heterogeneity across studies was evaluated with the Q test and I^2^ statistic (24). We defined an I^2^ value >50% as indicative of heterogeneity according to Cochrane Handbook. Stratified analysis was performed to identify the possible sources of heterogeneity based on living region (America/Europe vs. Asia), baseline age (<60 vs. ≥ 60, yr), gender (man vs. women), median duration of follow-up (≤9.3 vs. > 9.3, yr), cancer location (colon vs. rectum), quality scores (7 vs. 8–9), study design (prospective cohort vs. nested case-control/case-cohort), biomarker types (adipose vs. blood), and multiple adjustments (yes vs. no). A univariate meta-regression with restricted maximum likelihood was performed to measure if summary RR significantly differed between each stratum analyzed. Sensitivity analyses were performed to evaluate the possible influence of individual studies on the summary results. A possibility of publication bias was qualitatively delineated by the asymmetry of funnel plots and quantitatively evaluated by Egger's regression tests (25).

Dose-response meta-analyses were conducted to determine whether the levels of ALA in diet or circulating blood were dose-dependently associated with the risk of CRC. In brief, individual studies with three or more categories were included in the dose-response analysis and the median values of ALA levels in both diet and blood for each exposure category were assigned as previously described (26). A curvilinear trend was tested by using the methods previously described (27, 28). Specifically, restricted cubic splines with 3 knots (2 spline transformations) at fixed percentiles (25%, 50%, and 75%) were first created, and then a *P*-value for non-linearity was calculated to detect a potential departure from a simpler linear trend by testing the coefficient of the second spline equal to zero (29). In the presence of substantial linear trends (P for non-linearity > 0.05), a linear trend was estimated to achieve the association of per 1-g/day increment in dietary intake of ALA and per 0.1% increase in the levels of blood ALA with the risk of CRC by using a generalized least-squares regression (2-stage GLST in Stata) (27). Two-tailed *P* < 0.05 was considered statistically significant. Statistical analyses of all the data were performed by STATA version 15.1 (Stata CORP, College Station, TX).

## Results

**Figure 1:**
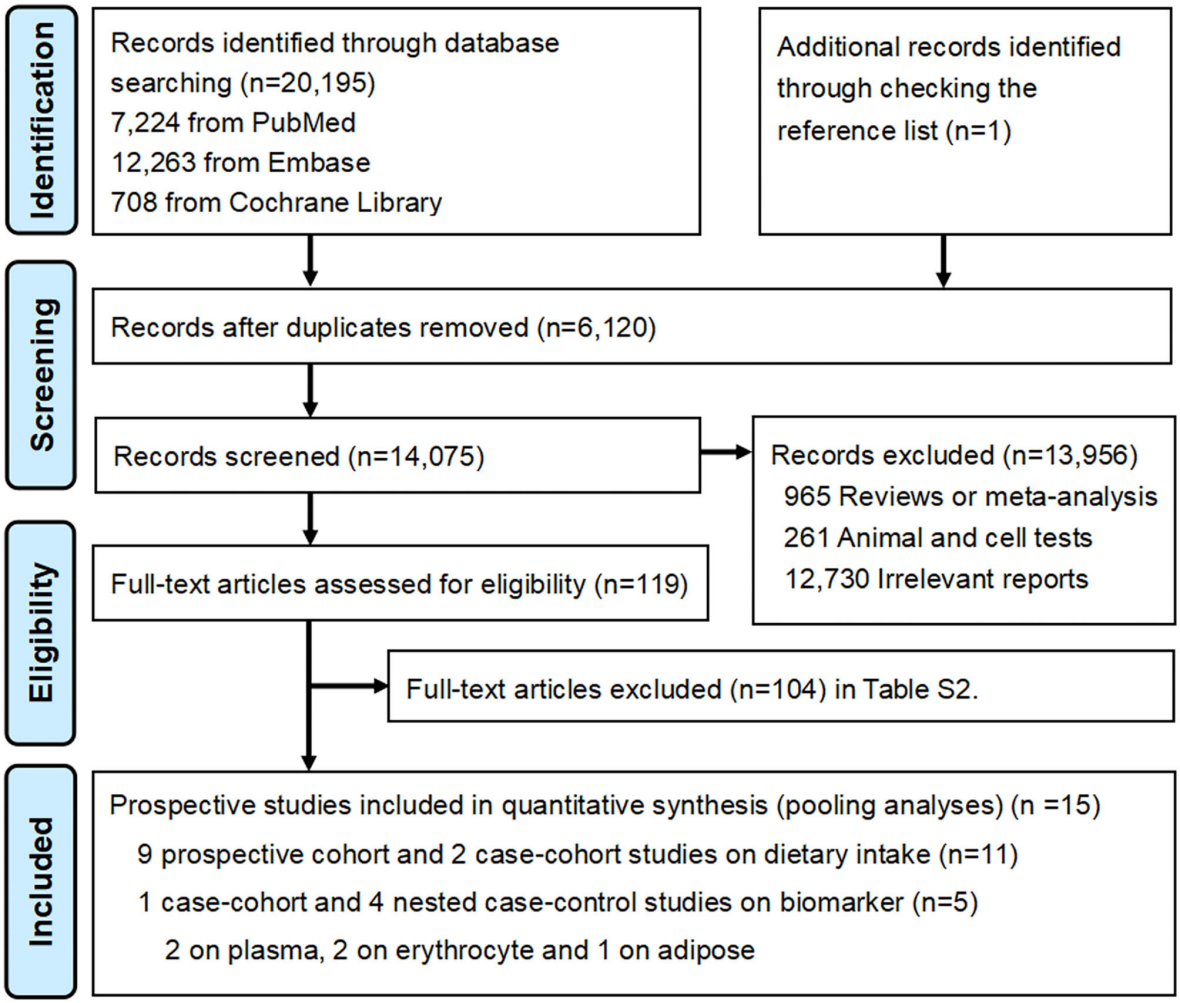
PRISMA Flow diagram for included prospective cohort studies.

The major result of the search strategy is presented in the PRISMA flow diagram (Figure 1). The initial search identified 20,196 records including 1 record through checking the reference list, from which 6,120 duplicates were removed. The remaining 14,075 records were screened for titles and abstracts. The preliminary screening left 119 potential articles, and subsequently, 104 articles were excluded for additional reasons after a full-text review (Supplementary Table 1). Finally, 15 prospective studies were eligible for the present meta-analysis, including 11 cohorts on dietary intake and 5 cohorts on biomarkers (four studies on blood and one study on adipose tissue).

### Baseline Characteristics

The characteristics of 15 independent prospective studies are presented in Table 1. During the follow-up duration of a 9.3-year median ranging from 1 to 28 years, 12,239 CRC cases were identified among 861,725 participants from the 15 prospective studies. Eleven cohorts of dietary intake of ALA were included, involving 10,583 cases and 854,818 participants in 9 prospective cohort studies (10, 30, 31, 33–36, 38, 39) and 2 case-cohort studies (32, 37), and dietary measurements were evaluated by food frequency questionnaires. For biomarkers of ALA, 5 prospective studies were included, involving 2,051 cases and 48,421 participants in 2 studies based on plasma (17, 37), 2 on erythrocyte (18, 40), and 1 on AT (16). ALA levels in different biospecimens were quantified by gas-liquid chromatography (GLC) and the measurement unit was set as a percentage, except for one study (μmol/L) (17). Both male and female were reported in five articles (17, 32, 37, 39, 40), only male in two articles (10, 30), only female in four articles (18, 31, 34, 38), and 4 articles separately reported male and female (16, 33, 35, 36). As for CRC locations, 13 articles reported total CRC (10, 16–18, 30, 31, 33–39), whereas 2 articles only separately reported colon cancer and rectal cancer (32, 40). Among all of the included studies, quality scores assessed by the 9-star NOS ranged from 7 to 9, with a median quality (≤7 stars) in 2 studies (36, 39) and high quality (≥ 8 stars) in 13 studies.

**Table 1:** Baseline characteristics of the individual prospective cohort studies.

**First author, published year**	**Location (cohort name)**	**Design**	**Cases/Participants**	**Age (median, yr), gender**	**Follow-up (median, yr)**	**Exposure of interest**		**Outcomes**		**QS**
						**Measurement**	**Exposure range (top vs. bottom)**	**Endpoints**	**RR (95% CI)**	
Pietinen et al. (30)	America (ATBCS)	PC	185/27,111	57.1, Male	8.0	Diet (FFQ)	Median of top quartile range vs. bottom in subjects: 2.4 vs. 1.0, g/day	CRC	1.40 (0.90, 2.10)	9
Terry et al. (31)	Europe	PC	460/61,463	52.0, Female	9.6	Diet (FFQ)	Median of top quartile range vs. bottom in subjects: 0.70 vs. 0.45, g/day	CRC	0.99 (0.75, 1.32)	8
								CC	0.90 (0.63, 1.28)	
								RC	1.11 (0.70, 1.78)	
Brink et al. (32)	Europe (NLCS)	CH	608/120,852	61.3, Both	4.4	Diet (FFQ)	Median of top quartile range vs. bottom in subjects: 1.8 vs. 0.70, g/day	CC	1.01 (0.75, 1.36)	9
								RC	0.91 (0.58, 1.44)	
Daniel et al. (33)	America (CPS-II)	PC	452/43,108	70.3, Male	6.0	Diet (FFQ)	Range of top quartile vs. bottom in subjects: ≥1.26 vs. <0.82, g/day	CRC	0.87 (0.66, 1.04)	9
		PC	417/55,972	68.5, Female	6.0	Diet (FFQ)	Range of top quartile vs. bottom in subjects: ≥1.19 vs. <0.78, g/day	CRC	1.38 (1.02, 1.85)	
Murff et al. (34)	Asia (SWHS)	PC	396/73,242	52.5, Female	9.0	Diet (FFQ)	Median of top quintile range vs. bottom in subjects: 1.44 vs. 0.58, g/day	CRC	1.16 (0.66, 2.06)	9
								CC	1.40 (0.58, 3.37)	
								RC	0.64 (0.22, 1.89)	
Sasazuki et al. (35)	Asia (JPHCS)	PC	774/41,382	56.9, Male	9.3	Diet (FFQ)	Median of top quintile range vs. bottom in subjects: 2.76 vs. 1.21, g/day	CC	0.84 (0.56, 1.28)	9
								RC	1.10 (0.61, 1.98)	
		PC	494/47,192	57.4, Female	9.3	Diet (FFQ)	Median of top quintile range vs. bottom in subjects: 2.64 vs. 1.35, g/day	CC	1.01 (0.65, 1.57)	
								RC	1.02 (0.50, 2.06)	
Song et al. (36)	America (NHS & HPFS)	PC	987/47,143	53.9, Male	20.6	Diet (FFQ)	Range of top quartile vs. bottom in subjects: ≥1.30 vs. <0.90, g/day	CRC	0.89 (0.70, 1.13)	7
								CC	0.96 (0.72, 1.30)	
								RC	0.68 (0.41, 1.15)	
		PC	1,469/76,386	50.4, Female	23.8	Diet (FFQ)	Range of top quartile vs. bottom in subjects: ≥1.20 vs. <0.90, g/day	CRC	1.05 (0.86, 1.29)	
								CC	1.09 (0.87, 1.37)	
								RC	0.84 (0.52, 1.37)	
Hodge et al. (37)	Europe (MCCS)	CH	395/41,514	58.5, Both	9.0	Diet (FFQ)	Range of top quintile vs. bottom in subjects: ≥1.13 vs. <0.66, g/day	CRC	1.09 (0.77, 1.53)	8
		CH	395/41,514	58.5, Both	9.0	Plasma (GLC)	Range of top quintile vs. bottom in subjects: ≥0.21 vs. <0.10, %	CRC	0.96 (0.69, 1.33)	
Shin et al. (38)	Europe (WLH Cohort)	PC	344/48,233	39.7, Female	21.3	Diet (FFQ)	Range of top quartile vs. bottom in subjects: 1.16-4.47 vs. 0.12-0.84, g/day	CRC	1.17 (0.86, 1.59)	9
								CC	0.96 (0.65, 1.41)	
								RC	1.61 (0.98, 2.69)	
Nguyen et al. (10)	Asia (SMHS)	PC	876/59,986	55.1, Male	9.8	Diet (FFQ)	Not available data	CRC	1.15 (0.92, 1.43)	9
								CC	0.98 (0.74, 1.31)	
								RC	1.45 (1.03, 2.05)	
Wan et al. (39)	America (NHS & HPFS)	PC	2,726/ 111,234	53.2, Both	24.3	Diet (FFQ)	Top quintile vs. bottom in subjects: not available	CRC	1.01 (0.90, 1.15)	7
Kojima et al. (16)	Asia (JACC Study)	NCC	83/324	60.5, Male	7.1	Adipose (GLC)	Range of top quartiles vs. bottom in subjects: >1.07 vs. <0.69, %	CRC	0.39 (0.16, 0.91)	9
			86/326	62.4, Female	7.1	Adipose (GLC)	Range of top quartile vs. bottom in subjects: >1.10 vs. <0.71, %	CRC	2.16 (0.87, 5.47)	
Cottet et al. (18)	Europe (E3N)	NCC	328/947	57.5, Female	9.0	Erythrocyte (GLC)	Range of top tertiles vs. bottom in subjects: >0.12 vs. <0.10, %	CRC	0.71 (0.49, 1.03)	8
Butler et al. (17)	Asia (SCHS)	NCC	350/700	59.7, Both	3.3	Plasma (GC-MS)	Range of top quartile vs. bottom in subjects: >3.8 vs. <1.9, umol/L	CC	0.41 (0.23, 0.73)	9
								RC	1.70 (0.84, 3.43)	
Wang et al. (40)	America (NHS & HPFS)	NCC	809/4,610	57.1, Both	20.0	Erythrocyte (GLC)	Per 1-SD change in subjects: 0.06, %	CRC	0.94 (0.88, 1.00)	9
								CC	0.94 (0.87, 1.02)	
								RC	0.94 (0.83, 1.06)	

*PC, prospective cohort; NCC, nested case-control; CH, case cohort; CRC, colorectal cancer; CC, colon cancer; RC, rectal cancer; RR, risk ratio; CI, confidence interval; SD, standard deviation; FFQ, food frequency questionnaire; GLC, gas-liquid chromatography; GC-MS, gas chromatography-mass spectrometry; QS, quality scores; ATBCS, Alpha-Tocopherol, Beta-Carotene Cancer Prevention Study; NCLS, The Netherlands Cohort Study; CPS, Cancer Prevention Study; SWHS, Shanghai Women's Health Study; JPHCS, Japan Public Health Center (JPHC)-Based Prospective Study; NHS, Nurses' Health Study; HPFS, Health Professionals Follow-up Study; MCCS, Melbourne Collaborative Cohort Study; WLH, Swedish Women's Lifestyle and Health; SMHS, Shanghai Men's Health Study; JACC, Japan Collaborative Cohort; E3N, Etude Epidémiologique auprès de femmes de la Mutuelle Générale de l'Education Nationale; SCHS, Singapore Chinese Health Study*.

### The Top Quartiles Vs. Bottom Analyses

**Figure 2:**
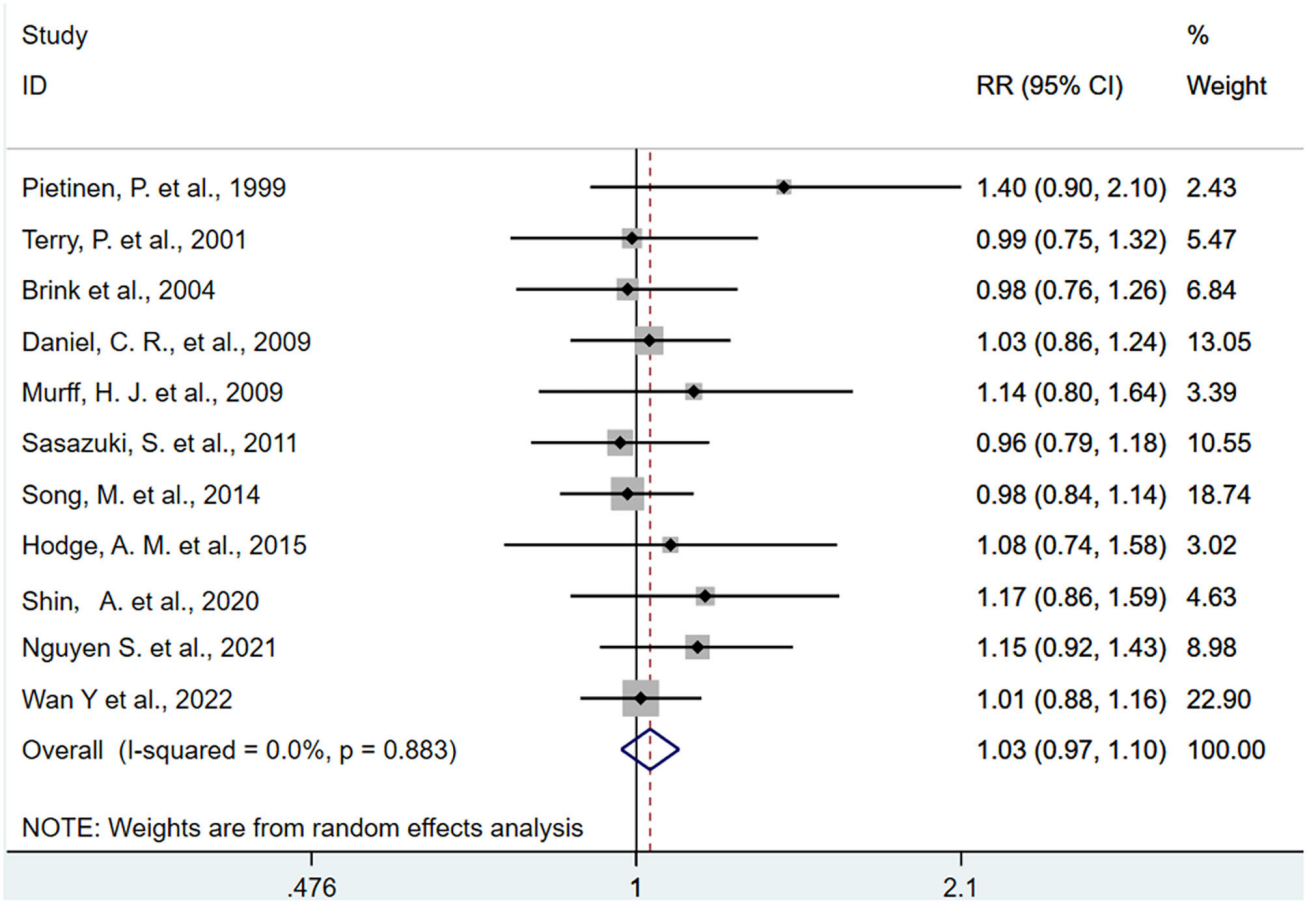
Forest plot of the association between dietary alpha-linolenic acid and risk of colorectal cancer in the top quartile compared with the bottom. Gray square represents RR in each original study, with square size reflecting the study-specific weight and the 95% confidence interval (CI) represented by horizontal bars. RRs from the individual study were pooled by random-effects model. The summary RR (SRR) and corresponding 95% CI were represented by the diamond. The degree of heterogeneity between individual studies was indicated by the I square statistic.

**Figure 3:**
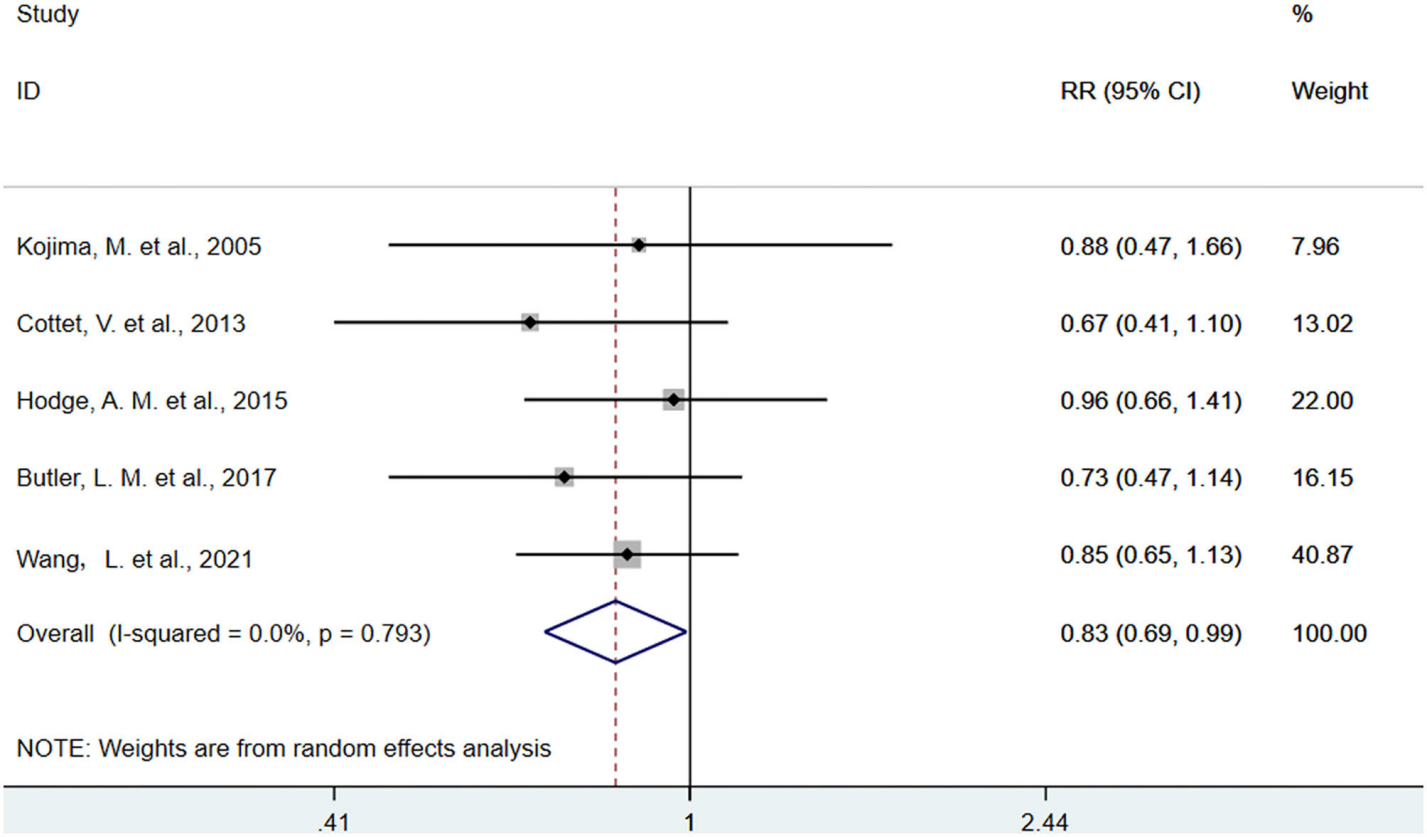
Forest plot of the association between biomarkers of alpha-linolenic acid and risk of colorectal cancer in the top quartile compared with the bottom. Gray square represents RR in each original study, with square size reflecting the study-specific weight and the 95% confidence interval (CI) represented by horizontal bars. RRs from the individual study were pooled by random-effects model. The summary RR (SRR) and corresponding 95% CI were represented by the diamond. The degree of heterogeneity between individual studies was indicated by the I square statistic.

The pooled association comparing the top with the bottom quartiles of dietary intake and biomarkers of ALA were presented in Figures 2, 3. The SRR for ALA in diet was 1.03 (95% CI: 0.97, 1.10), with no between-study heterogeneity (I^2^ = 0.00%). An inverse association was found between ALA biomarker and CRC (SRR = 0.83, 95%CI: 0.69, 0.99), with no between-study heterogeneity (I^2^ = 0.00%).

In the stratified analysis of dietary ALA intake concerning CRC (Supplementary Table 4), there was no evidence that the estimated summary RR differed significantly by living regions, age, gender, follow-up duration, cancer location, quality scores, study design, and multiple adjustments. In stratified analyses for the biomarker of ALA (Supplementary Table 5), increased levels of ALA in biospecimens were more pronounced with decreased risk of CRC in middle-aged persons (SRR = 0.83, 95%CI: 0.69, 0.99) but not in elderly persons (SRR = 0.88, 95%CI: 0.47, 1.65), while the difference between the two populations cannot be tested with a meta-regression. As for different types of biomarkers, although the pooled associations for circulating levels of ALA (SRR = 0.83, 95% CI: 0.69, 0.99) were found to be more apparent than that for AT (SRR = 0.88, 95% CI: 0.47, 1.65), results of meta-regression did not show a statistically significant difference between the two biomarkers.

In sensitivity analyses that exclude one study at a time and reanalyzed the remaining data, the exclusion of any individual study of ALA in the diet as interest exposure did not substantially change the summary result (Supplementary Figure 1). As for biomarkers, results of sensitivity analyses found that the overall summary associations were modestly changed when one study by Cottet V et al. (18) was omitted, with the SRR ranging from 0.83 (0.69, 0.98) to 0.86 (0.72, 1.02) (Supplementary Figure 2).

In publication bias analyses for either dietary intake or biomarker, no publication bias was indicated by Begg's funnel plot (P for bias of dietary intake = 0.06, P for bias of biomarker = 0.46) (Supplementary Figures 3, 4) or Egger's regression test (P for bias of dietary intake = 0.06, P for bias of dietary intake = 0.55) (Supplementary Figures 5, 6).

### Dose-Response Analyses

**Figure 4:**
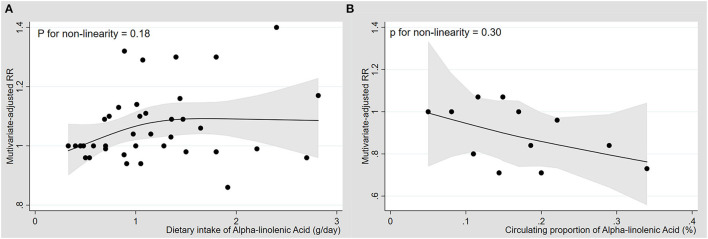
Dose-response association between diet and blood levels of alpha-linolenic acid and risk of colorectal cancer. Multivariate-adjusted relative risks (RRs) from all categories of ALA levels in diet or blood in each original study were represented by the small black circle. The corresponding non-linear dose-response relationships of dietary **(A)** and blood levels of ALA **(B)** with the risk of colorectal cancer were assessed by a restricted cubic spline model with three fixed knots and represented by the black solid line, respectively.

Nine cohorts with dietary intake of ALA were available for the dose-response analyses (30–38). There was no significantly curvilinear relationship with the CRC risk (Figure 4A), and the association was not statistically significant in the linear model with per 1.0-g/d ALA increase (p for linearity = 0.22) (Supplementary Figure 7).

Four cohorts with blood levels of ALA were available for the dose-response analyses (17, 18, 37, 40), and there was no significantly curvilinear relationship with a test for non-linearity (Figure 4B). The levels of ALA in blood had a linear dose-response association with CRC (p for linearity = 0.04), and each 0.1% increase in ALA levels resulted in a 10% reduction of risk of CRC (SRR= 0.90, 95%CI: 0.81, 0.99; I^2^ = 35.9%) (Supplementary Figure 8).

## Discussion

To the best of our knowledge, this present study is the first meta-analysis that specially focused on the impacts of plant-based n-3 ALA (diet vs. biomarker) on the risk of CRC. Our pooled analysis of prospective cohorts suggested that blood levels of ALA were linearly and inversely associated with CRC risk, but no significant association was found for dietary intake of ALA. Such findings support that increased levels of ALA intake have potential benefits in preventing the development of CRC, which may further extend the previous meta-analyses with mixed n-3 PUFAs as interest exposure to highlight that the plant-derived ALA remains a protective nutrient for the incident CRC (9, 10, 15).

Results of our meta-analysis based on the prospective cohorts with the dietary estimation of AL showed a null association with CRC risk, and there was no significant difference by age, gender, geographical regions, cancer locations, duration of follow-up, or multiple adjustments. Compared with our present study, most of the previous studies especially focused on food n-3 PUFAs mixed plant- with marine-based sources (9, 10). The summary evidence for especially focusing on the association between ALA intake (plant n-3 fatty acids) and CRC risk was currently limited. Nevertheless, our observation of the null findings for dietary ALA intake was consistent with the previous results in three publications of meta-analytic reviews with mixed n-3 PUFA as interest exposures. Of note, in population-based food investigation, measurement error and bias always occurred during the performance of dietary assessment using the food frequency questionnaire, which may have changed the direction of the observed associations. Fatty acids are especially prone to this misclassification of dietary intake because similar foods may have different PUFA compositions that are difficult to be distinguished by using food descriptions in the questionnaire tools. Another possibility was that measurement errors in assessing individual fatty acid intake may have attenuated the beneficial association with ALA intake toward a null. Third, although direct evidence in laboratory studies proved that the plant-derived ALA may suppress the development of CRC through downregulation of malignant in human and mouse colon cancer cells, the dosage of n-3 PUFA used in animal studies is much higher than the daily intake of ALA in humans (41). Therefore, it is possible that in the normal range of the human diet, it cannot be concluded that there is a protective effect of dietary intake of plant-based n-3 PUFA on the development of CRC.

PUFA levels in human tissue (e.g., blood or AT) are currently regarded as a reasonable biological marker of habitual dietary fat intake, with sufficient evidence in the strong correlation between dietary fatty acid intake and circulating levels even if it is a single blood sample. Results of our meta-analysis based on five prospective cohorts revealed that increased levels of ALA in biospecimen (blood and AT) were significantly associated with a reduced risk of CRC. In support of these major findings, a similar inverse association with biomarker ALA was also observed in two publications of population-based epidemiological studies (16, 17). Nevertheless, the perfect associations did not reach a statistical significance in most of the previous prospective studies including a recent meta-analytic review (15, 18, 32, 37). One possible explanation was that the results of the prior meta-analysis could probably be affected by a limited number of included studies (only three cohorts with blood PUFAs), thereby perhaps leading to insufficient statistical power. Compared with the recent publication of meta-analysis, available data on different biomarkers of ALA (serum/plasm/erythrocyte/AT) from more comprehensive cohorts including recent literature of erythrocyte measurements in a larger number of 4,517 participants and another research on AT measurement were pooled in the present study (16, 40), which help enhance the statistical power to update the previous summary evidence. Moreover, multivariate-adjusted RR for the highest vs. the lowest category from each eligible study was transformed to involve comparisons between the highest and the lowest quartiles of baseline ALA levels, which may have greatly minimized statistical heterogeneity to achieve the reliability of our summary results. Finally, our findings based on dose-response meta-analyses with a test for linearity or non-linearity showed that decreased risk of CRC is linearly related to increased levels of ALA in blood, which may reinforce the robustness in association with biomarkers.

In the stratified analysis of biomarker ALA in relation to CRC, we found a beneficial association estimation in males rather than in females. Given that estrogen might have participated in the etiology of CRC (42, 43), losing adjustments for menopausal status and hormone therapy drugs might have lowered the ability to test the preferred effects in females. However, the results of the meta-analysis with interaction tests did not detect the gender-based difference. Moreover, a negative association was found to be more significant in elderly persons than in elderly persons, but the difference between the two populations cannot be tested with meta-regression analyses. It is noted that the elderly individuals seem to have more commodities with the obesity-related metabolic disorder such as dyslipidemia than young persons, which may have additionally increased the initiation and progression of CRC (44). When further stratified by biomarker types, we found that the lower risk of CRC was linearly associated with blood levels of ALA but not with AT-based biomarkers. Most of the observational studies measured fatty acid profiles in AT that can mostly represent triacylglycerol to mirror a relative long-term intake (over 2 years), this tissue does not seem to be a perfect biomarker of n-3 PUFA intake due to relatively low incorporation of ALA in AT (45). One cohort of AT measurement only was eligible for the current study, which could greatly minimize the possibility of generalizable results for all persons. Of note, erythrocyte levels mostly represent membrane PL to indicate a medium-term FA intake (several months) than blood lipids in plasma/serum indicating PUFAs' concentrations over recent days and cannot be easily affected by the postprandial status of the individual (46). Therefore, the summary estimates based on prospective cohorts with blood measurements are more reliable to diet-related evidence in elucidating the causal relationship with ALA. However, our observation of erythrocyte-based biomarkers had a marginally significant association with risk of CRC, which may have in part or at least attenuated our ultimate findings. Moreover, given that the limited number of eligible articles in each stratum analyzed might have diminished statistical power, such findings based on each subgroup need to be interpreted with caution and requires future confirmation in more large-scale cohorts at biomarker levels.

There are several biological mechanisms underlying the protective effect of ALA on the development of CRC. First, ALA is an essential precursor of long-chain n-3 fatty acids *in vivo*, which can be progressively transferred to eicosapentaenoic acid (EPA), docosapentaenoic acid (DPA), and finally docosahexaenoic acid (DHA) (47), through an extremely low-conversion rate (48). ALA might have a potential inhibitive effect on the CRC development by the limited transformation to marine n-3 PUFAs (49). In addition, as a plant-derived member of food n-3 fatty acids, ALA could modulate the activity of cyclooxygenases (COX) and inhibit tumor growth by reducing n-6 PUFA-derived 2-series prostaglandin (PGE2) and promoting n-3 family derived 3-series prostaglandin (PGE3) (50, 51). Third, ALA dampened the inflammatory phenotype of M1-like macrophages, thereby reducing the expression levels of pro-inflammatory markers such as IL-6, IL-1β, TNF-α, and MCP-1 in human THP-1 cells (52). Finally, ALA might have individually regulated the apoptosis mechanism and NF-κB signaling pathway related with inflammatory response to control tumor proliferations, migrations, and invasions (41, 53, 54).

Several strengths are currently emphasized in our study. The eligible prospective cohort studies only were included, and therefore no recall and selection bias caused by retrospective studies would influence the summary result. Besides, stratified analyses with a meta-regression test indicated that the overall association estimations were not affected by the strata analyzed such as age, follow-up years, cancer location, and multiple adjustments, thereby increasing the potential possibility of the robust performance of final results. Third, compared with previous meta-analyses (10, 15), we included many published cohorts to update the previous summary evidence, which helps to enhance statistical power. Fourth, because of report bias in dietary measurements of fatty acids, we summarized the evidence on biomarker levels in the blood or AT, thereby increasing the stable generalizability of findings. Finally, no significant publication bias or between-study heterogeneity may have greatly enhanced the reliability of the summary result in the present study.

There are also several limitations in the present study. First, sensitivity analysis for biomarkers ALA indicated that exclusion of one study would potentially change the direction of the overall result toward a null (18). However, this study enrolled volunteers from a selected population of highly educated women, which was not representative of the general population. We therefore cannot rule out the possibility that selection bias might have seriously affected the association estimated in this study. Second, RRs (HRs) for various category levels in each original study were transformed with the top vs. bottom quartiles to provide a consistent approach to the meta-analysis, in which systematic error might have occurred during the data transformation. Third, though each original study controlled multiple confounding factors, there were still some residual confounders that might have changed the direction of the summary association. Fourth, though the beneficial association for the biomarker of ALA was found to be more significant in male and middle-aged populations, the results based on the subgroup analyses may not be popularized because of the limited number of included studies. Fifth, dietary changes or changes in food compositions may have occurred after blood collections and before the onset of CRC, perhaps leading to an underestimation of the pooled association. Sixth, misclassification in dietary estimations is inevitable, which was likely to bias the pooled association toward a null. Finally, we found a significant inverse association for ALA levels in the blood, but the results of AT measurement in relation to CRC risk are needed to be interpreted with more caution because of the limited number of published cohorts.

## Conclusion

The current meta-analysis indicated that biomarkers of ALA were inversely associated with the incident CRC, and each 0.1% increase in circulating levels of ALA was associated with 10% reduction in CRC risk. Encouraging the consumption of foods rich in ALA to improve its levels in the blood may potentially decrease the risk of CRC. Nevertheless, well-designed and large-scale cohorts with biomarkers are still needed for better reconfirming the potential impacts of ALA intake in the primary prevention of CRC.

## Data Availability Statement

The original contributions presented in the study are included in the article/Supplementary Material, further inquiries can be directed to the corresponding authors.

## Author Contributions

BY conceived the idea and designed the study strategy and provided critical revisions of the manuscript for important intellectual content, administrative and funding support, and supervision. Z-BD, Y-LX, YT, and B-BH conducted a reference search. Z-BD summarized the data and conducted data acquisition and statistical analyses. Z-BD and X-LR drafted the manuscript. All authors contributed to the article and approved the submitted version.

## Funding

This work is supported by the Programs Foundation of the Wenzhou Medical University of Zhejiang Province, China (89217015), the Scientific Technician Funding of Wenzhou Science and Technology Bureau (X20210055), the Basic Medicine Funding of Wenzhou Science and Technology Bureau (Y20180195), and by the 2017 Chinese Nutrition Society (CNS) Nutrition Research Foundation—DSM Research Fund (95017008). The funders have no role in study design, data collection and analysis, decision to publish, or preparation of the manuscript.

## Conflict of Interest

The authors declare that the research was conducted in the absence of any commercial or financial relationships that could be construed as a potential conflict of interest.

## Publisher's Note

All claims expressed in this article are solely those of the authors and do not necessarily represent those of their affiliated organizations, or those of the publisher, the editors and the reviewers. Any product that may be evaluated in this article, or claim that may be made by its manufacturer, is not guaranteed or endorsed by the publisher.

## References

[1] Dekker E Tanis PJ Vleugels JLA Kasi PM Wallace MB. Colorectal cancer. Lancet. (2019) 394:1467–80. 10.1016/S0140-6736(19)32319-0 31631858

[2] Arnold M Sierra MS Laversanne M Soerjomataram I Jemal A Bray F. Global patterns and trends in colorectal cancer incidence and mortality. Gut. (2017) 66:683–91. 10.1136/gutjnl-2015-310912 26818619

[3] Yang J Yu J. The association of diet, gut microbiota and colorectal cancer: what we eat may imply what we get. Protein Cell. (2018) 9:474–87. 10.1007/s13238-018-0543-6 29713943 PMC5960467

[4] Mocellin MC Camargo CQ Nunes EA Fiates GMR Trindade E. A systematic review and meta-analysis of the n-3 polyunsaturated fatty acids effects on inflammatory markers in colorectal cancer. Clin Nutr. (2016) 35:359–69. 10.1016/j.clnu.2015.04.013 25982417

[5] Yuan Q Xie F Huang W Hu M Yan Q Chen Z. The review of alpha-linolenic acid: Sources, metabolism, and pharmacology. Phytother Res. (2022) 36:164–88. 10.1002/ptr.7295 34553434

[6] Caligiuri SP Aukema HM Ravandi A Guzman R Dibrov E Pierce GN. Flaxseed consumption reduces blood pressure in patients with hypertension by altering circulating oxylipins via an alpha-linolenic acid-induced inhibition of soluble epoxide hydrolase. Hypertension. (2014) 64:53–9. 10.1161/HYPERTENSIONAHA.114.03179 24777981

[7] Gomes PM Hollanda-Miranda WR Beraldo RA Castro AV Geloneze B Foss MC. Supplementation of alpha-linolenic acid improves serum adiponectin levels and insulin sensitivity in patients with type 2 diabetes. Nutrition. (2015) 31:853–7. 10.1016/j.nut.2014.12.028 25933493

[8] Schiessel DL Yamazaki RK Kryczyk M Coelho I Yamaguchi AA Pequito DC. alpha-linolenic fatty acid supplementation decreases tumor growth and cachexia parameters in walker 256 tumor-bearing rats. Nutr Cancer. (2015) 67:839–46. 10.1080/01635581.2015.1043021 26011096

[9] Liu J Li X Hou J Sun J Guo N Wang Z. Dietary intake of N-3 and N-6 polyunsaturated fatty acids and risk of cancer: meta-analysis of data from 32 studies. Nutr Cancer. (2021) 73:901–13. 10.1080/01635581.2020.1779321 32530319

[10] Nguyen S Li H Yu D Cai H Gao J Gao Y. Dietary fatty acids and colorectal cancer risk in men: a report from the Shanghai Men's Health Study and a meta-analysis. Int J Cancer. (2021) 148:77–89. 10.1002/ijc.33196 32638381 PMC11067784

[11] Bingham SA Luben R Welch A Wareham N Khaw KT Day N. Are imprecise methods obscuring a relation between fat and breast cancer? Lancet. (2003) 362:212–4. 10.1016/S0140-6736(03)13913-X 12885485

[12] Hosomi K Kiyono H Kunisawa J. Fatty acid metabolism in the host and commensal bacteria for the control of intestinal immune responses and diseases. Gut Microbes. (2020) 11:276–84. 10.1080/19490976.2019.1612662 31120334 PMC7524326

[13] Clay SL Fonseca-Pereira D Garrett WS. Colorectal cancer: the facts in the case of the microbiota. J Clin Invest. (2022) 132:e155101. 10.1172/JCI155101 35166235 PMC8843708

[14] Burdge GC. Metabolism of alpha-linolenic acid in humans. Prostaglandins Leukot Essent Fatty Acids. (2006) 75:161–8. 10.1016/j.plefa.2006.05.013 16828546

[15] Kim Y Kim J. Intake or blood levels of n-3 polyunsaturated fatty acids and risk of colorectal cancer: a systematic review and meta-analysis of prospective studies. Cancer Epidemiol Biomarkers Prev. (2020) 29:288–99. 10.1158/1055-9965.EPI-19-0931 31767566

[16] Kojima M Wakai K Tokudome S Suzuki K Tamakoshi K Watanabe Y. Serum levels of polyunsaturated fatty acids and risk of colorectal cancer: a prospective study. Am J Epidemiol. (2005) 161:462–71. 10.1093/aje/kwi066 15718482

[17] Butler LM Yuan JM Huang JY Su J Wang R Koh WP. Plasma fatty acids and risk of colon and rectal cancers in the Singapore Chinese Health Study. NPJ precision oncology. (2017) 1:38. 10.1038/s41698-017-0040-z 29872717 PMC5871823

[18] Cottet V Collin M Gross AS Boutron-Ruault MC Morois S Clavel-Chapelon F. Erythrocyte membrane phospholipid fatty acid concentrations and risk of colorectal adenomas: a case-control nested in the french E3N-EPIC cohort study. Cancer Epidemiol Biomarkers Prev. (2013) 22:1417–27. 10.1158/1055-9965.EPI-13-0168 23704475

[19] Stroup DF Berlin JA Morton SC Olkin I Williamson GD Rennie D. Meta-analysis of observational studies in epidemiology: a proposal for reporting. Meta-Analysis Of Observational Studies in Epidemiology (MOOSE) group. JAMA. (2000) 283:2008–12. 10.1001/jama.283.15.2008 10789670

[20] Wells GA, Shea, B, O'Connell Peterson, J, Welch, V, Losos, M, . The Newcastle-Ottawa Scale (NOS) for Assessing the Quality of Nonrandomised Studies in Meta-Analyses. (2011). Available online at: www.ohri.ca/programs/clinical_epidemiology/oxford.htm (accessed November 21, 2012).

[21] Chene G Thompson SG. Methods for summarizing the risk associations of quantitative variables in epidemiologic studies in a consistent form. Am J Epidemiol. (1996) 144:610–21. 10.1093/oxfordjournals.aje.a008971 8797521

[22] Danesh J Collins R Appleby P Peto R. Association of fibrinogen, C-reactive protein, albumin, or leukocyte count with coronary heart disease: meta-analyses of prospective studies. JAMA. (1998) 279:1477–82. 10.1001/jama.279.18.1477 9600484

[23] DerSimonian R Laird N. Meta-analysis in clinical trials. Control Clin Trials. (1986) 7:177–88. 10.1016/0197-2456(86)90046-2 3802833

[24] Higgins JP Thompson SG Deeks JJ Altman DG. Measuring inconsistency in meta-analyses. BMJ. (2003) 327:557–60. 10.1136/bmj.327.7414.557 12958120 PMC192859

[25] Egger M Davey Smith G Schneider M Minder C. Bias in meta-analysis detected by a simple, graphical test. BMJ. (1997) 315:629–34. 10.1136/bmj.315.7109.629 9310563 PMC2127453

[26] Thesing CS Lamers F Bot M Penninx B Milaneschi Y. Response to “International Society for Nutritional Psychiatry Research Practice Guidelines for Omega-3 Fatty Acids in the Treatment of Major Depressive Disorder” by Guu et al. (2019). Psychother Psychosom. (2020) 89:48. 10.1159/000504100 31655818 PMC7050662

[27] Orsini N BR Greenland S. Generalized least squares for trend estimation of summarized dose-response data. Stata J. (2006) 6:40–57. 10.1177/1536867X0600600103

[28] Jackson D White IR Thompson SG. Extending DerSimonian and Laird's methodology to perform multivariate random effects meta-analyses. Stat Med. (2010) 29:1282–97. 10.1002/sim.3602 19408255

[29] Orsini N Li R Wolk A Khudyakov P Spiegelman D. Meta-analysis for linear and nonlinear dose-response relations: examples, an evaluation of approximations, and software. Am J Epidemiol. (2012) 175:66–73. 10.1093/aje/kwr265 22135359 PMC3244608

[30] Pietinen P Malila N Virtanen M Hartman TJ Tangrea JA Albanes D. Diet and risk of colorectal cancer in a cohort of Finnish men. CCC. (1999) 10:387–96. 10.1023/A:1008962219408 10530608

[31] Terry P Bergkvist L Holmberg L Wolk A. No association between fat and fatty acids intake and risk of colorectal cancer. Cancer Epidemiol Biomarkers Prev. (2001) 10:913–4. Available online at: http://cebp.aacrjournals.org/content/10/8/913 11489762

[32] Brink M Weijenberg MP De Goeij AF Schouten LJ Koedijk FD Roemen GM. Fat and K-ras mutations in sporadic colorectal cancer in The Netherlands Cohort Study. Carcinogenesis. (2004) 25:1619–28. 10.1093/carcin/bgh177 15117813

[33] Daniel CR McCullough ML Patel RC Jacobs EJ Flanders WD Thun MJ. Dietary intake of ω-6 and ω-3 fatty acids and risk of colorectal cancer in a prospective cohort of U. S men and women. Cancer Epidemiol Biomarkers Prev. (2009) 18:516–25. 10.1158/1055-9965.EPI-08-0750 19190143

[34] Murff HJ Shu XO Li H Dai Q Kallianpur A Yang G. A prospective study of dietary polyunsaturated fatty acids and colorectal cancer risk in Chinese women. Cancer Epidemiol Biomarkers Prev. (2009) 18:2283–91. 10.1158/1055-9965.EPI-08-1196 19661088 PMC2731694

[35] Sasazuki S Inoue M Iwasaki M Sawada N Shimazu T Yamaji T. Intake of n-3 and n-6 polyunsaturated fatty acids and development of colorectal cancer by subsite: Japan Public Health Center-based prospective study. Int J Cancer. (2011) 129:1718–29. 10.1002/ijc.25802 21120874

[36] Song M Chan AT Fuchs CS Ogino S Hu FB Mozaffarian D. Dietary intake of fish, ω-3 and ω-6 fatty acids and risk of colorectal cancer: A prospective study in U. S men and women. Int J Cancer. (2014) 135:2413–23. 10.1002/ijc.28878 24706410 PMC4159425

[37] Hodge AM Williamson EJ Bassett JK MacInnis RJ Giles GG English DR. Dietary and biomarker estimates of fatty acids and risk of colorectal cancer. Int J Cancer. (2015) 137:1224–34. 10.1002/ijc.29479 25683336

[38] Shin A Cho S Sandin S Lof M Oh MY Weiderpass E. Omega-3 and−6 fatty acid intake and colorectal cancer risk in swedish women's lifestyle and health cohort. Cancer Res Treatment. (2020) 52:848–54. 10.4143/crt.2019.550 32138465 PMC7373878

[39] Wan Y Wu K Wang L Yin K Song M Giovannucci EL. Dietary fat and fatty acids in relation to risk of colorectal cancer. Eur J Nutr. (2022). 10.1007/s00394-021-02777-9 35048194

[40] Wang L Hang D He X Lo CH Wu K Chan AT. A prospective study of erythrocyte polyunsaturated fatty acids and risk of colorectal serrated polyps and conventional adenomas. Int J Cancer. (2021) 148:57–66. 10.1002/ijc.33190 32638350 PMC7722098

[41] Chamberland JP Moon HS. Down-regulation of malignant potential by alpha linolenic acid in human and mouse colon cancer cells. Fam Cancer. (2015) 14:25–30. 10.1007/s10689-014-9762-z 25336096

[42] Tamakoshi K Wakai K Kojima M Watanabe Y Hayakawa N Toyoshima H. A prospective study on the possible association between having children and colon cancer risk: findings from the JACC Study. Cancer Sci. (2004) 95:243–7. 10.1111/j.1349-7006.2004.tb02210.x 15016324 PMC11159313

[43] Mason JK Kharotia S Wiggins AK Kitson AP Chen J Bazinet RP. 17beta-estradiol increases liver and serum docosahexaenoic acid in mice fed varying levels of alpha-linolenic acid. Lipids. (2014) 49:745–56. 10.1007/s11745-014-3913-8 24913495

[44] Yarla N Madka V Rao C. Targeting triglyceride metabolism for colorectal cancer prevention and therapy. Curr Drug Targets. (2021). 10.2174/1389450122666210824150012 34431463

[45] Giuliani A Ferrara F Scimo M Angelico F Olivieri L Basso L. Adipose tissue fatty acid composition and colon cancer: a case-control study. Eur J Nutr. (2014) 53:1029–37. 10.1007/s00394-013-0605-8 24178365

[46] Arab L Akbar J. Biomarkers and the measurement of fatty acids. Public Health Nutr. (2002) 5(6A):865–71. 10.1079/PHN2002391 12638594

[47] Barcelo-Coblijn G Murphy EJ. Alpha-linolenic acid and its conversion to longer chain n-3 fatty acids: benefits for human health and a role in maintaining tissue n-3 fatty acid levels. Prog Lipid Res. (2009) 48:355–74. 10.1016/j.plipres.2009.07.002 19619583

[48] Burns-Whitmore B Froyen E Heskey C Parker T San Pablo G. Alpha-Linolenic and linoleic fatty acids in the vegan diet: do they require dietary reference intake/adequate intake special consideration? Nutrients. (2019) 11:2365. 10.3390/nu11102365 31590264 PMC6835948

[49] Yang B Wang FL Ren XL Li D. Biospecimen long-chain N-3 PUFA and risk of colorectal cancer: a meta-analysis of data from 60,627 individuals. PLoS ONE. (2014) 9:e110574. 10.1371/journal.pone.0110574 25375637 PMC4222788

[50] Hopkins GJ Kennedy TG Carroll KK. Polyunsaturated fatty acids as promoters of mammary carcinogenesis induced in Sprague-Dawley rats by 7,12-dimethylbenz[a]anthracene. J Natl Cancer Inst. (1981) 66:517–22. 6782319

[51] Poole EM Bigler J Whitton J Sibert JG Kulmacz RJ Potter JD. Genetic variability in prostaglandin synthesis, fish intake and risk of colorectal polyps. Carcinogenesis. (2007) 28:1259–63. 10.1093/carcin/bgm026 17277229

[52] Pauls SD Rodway LA Winter T Taylor CG Zahradka P Aukema HM. Anti-inflammatory effects of alpha-linolenic acid in M1-like macrophages are associated with enhanced production of oxylipins from alpha-linolenic and linoleic acid. J Nutr Biochem. (2018) 57:121–9. 10.1016/j.jnutbio.2018.03.020 29698923

[53] Hassan A Ibrahim A Mbodji K Coeffier M Ziegler F Bounoure F. An alpha-linolenic acid-rich formula reduces oxidative stress and inflammation by regulating NF-kappaB in rats with TNBS-induced colitis. J Nutr. (2010) 140:1714–21. 10.3945/jn.109.119768 20724486

[54] Gonzalez-Fernandez MJ Ortea I Guil-Guerrero JL. alpha-Linolenic and gamma-linolenic acids exercise differential antitumor effects on HT-29 human colorectal cancer cells. Toxicol Res (Camb). (2020) 9:474–83. 10.1093/toxres/tfaa046 32905142 PMC7467275

