# Supplementary material for: Association of Dietary Intake and Biomarker of α-Linolenic Acid With Incident Colorectal Cancer: A Dose-Response Meta-Analysis of Prospective Cohort Studies
Source: Front Nutr. 2022 Jul 7;9:948604. doi: 10.3389/fnut.2022.948604 (PMC9301188; doi:10.3389/fnut.2022.948604)
Supplement: Supplementary file 1 [file Data_Sheet_1.docx]

Supplementary Material

# Supplementary Data

## Literature searching strategies

Literature search strategy in Pubmed: 7224 (publication till 2022 Feb):

(("Colorectal Neoplasms"[Mesh]) OR (Colonic Neoplasm[Title/Abstract]) OR (Neoplasm, Colonic[Title/Abstract]) OR (Neoplasms, Colonic[Title/Abstract]) OR (Colon Neoplasms[Title/Abstract]) OR (Colon Neoplasm[Title/Abstract]) OR (Neoplasm, Colon[Title/Abstract]) OR (Cancer of Colon[Title/Abstract]) OR (Neoplasms, Colon[Title/Abstract]) OR (Colon Cancers[Title/Abstract]) OR (Cancer of the Colon[Title/Abstract]) OR (Colonic Cancer[Title/Abstract]) OR (Cancer, Colonic[Title/Abstract]) OR (Cancers, Colonic[Title/Abstract]) OR (Colonic Cancers[Title/Abstract]) OR (Colon Cancer[Title/Abstract]) OR (Cancer, Colon[Title/Abstract]) OR (Cancers, Colon[Title/Abstract]) OR (Colonic Neoplasms[Title/Abstract]) OR (Rectal Neoplasms[Title/Abstract]) OR (Neoplasm, Rectal[Title/Abstract]) OR (Rectal Neoplasm[Title/Abstract]) OR (Rectum Neoplasms[Title/Abstract]) OR (Neoplasm, Rectum[Title/Abstract]) OR (Rectum Neoplasm[Title/Abstract]) OR (Rectal Tumors[Title/Abstract]) OR (Rectal Tumor[Title/Abstract]) OR (Tumor, Rectal[Title/Abstract]) OR (Neoplasms, Rectal[Title/Abstract]) OR (Cancer of Rectum[Title/Abstract]) OR (Rectum Cancers[Title/Abstract]) OR (Rectal Cancer[Title/Abstract]) OR (Cancer, Rectal[Title/Abstract]) OR (Rectal Cancers[Title/Abstract]) OR (Rectum Cancer[Title/Abstract]) OR (Cancer, Rectum[Title/Abstract]) OR (Cancer of the Rectum[Title/Abstract]) OR (Colorectal Neoplasms[Title/Abstract]) OR (Colorectal Neoplasm[Title/Abstract]) OR (Neoplasm, Colorectal[Title/Abstract]) OR (Neoplasms, Colorectal[Title/Abstract]) OR (Colorectal Tumors[Title/Abstract]) OR (Colorectal Tumor[Title/Abstract]) OR (Tumor, Colorectal[Title/Abstract]) OR (Tumors, Colorectal[Title/Abstract]) OR (Colorectal Cancer[Title/Abstract]) OR (Cancer, Colorectal[Title/Abstract]) OR (Cancers, Colorectal[Title/Abstract]) OR (Colorectal Cancers[Title/Abstract]) OR (Colorectal Carcinoma[Title/Abstract]) OR (Carcinoma, Colorectal[Title/Abstract]) OR (Carcinomas, Colorectal[Title/Abstract]) OR (Colorectal Carcinomas[Title/Abstract])) AND (("Fatty Acids"[Mesh]) OR (Fatty Acids[Title/Abstract]) OR (Fatty Acid[Title/Abstract]) OR (Fatty Acids, Esterified[Title/Abstract]) OR (Esterified Fatty Acids[Title/Abstract]) OR (Esterified Fatty Acid[Title/Abstract]) OR (Acid, Esterified Fatty[Title/Abstract]) OR (Fatty Acid, Esterified[Title/Abstract]) OR (Fatty Acids, Saturated[Title/Abstract]) OR (Saturated Fatty Acids[Title/Abstract]) OR (Saturated Fatty Acid[Title/Abstract]) OR (Acid, Saturated Fatty[Title/Abstract]) OR (Fatty Acid, Saturated[Title/Abstract]) OR (Aliphatic Acids[Title/Abstract]) OR (Linolenic acid[Title/Abstract]) OR (Eicosapentaenoic acid[Title/Abstract]) OR (Docosapentaenoic acid[Title/Abstract]) OR (Docosahexaenoic acid[Title/Abstract]) OR (Linoleic acid[Title/Abstract]) OR (arachidonic acid[Title/Abstract]) OR (fat[Title/Abstract]))

Literature search strategy in Embase: 12263 (publication till 2022 Feb):

#1. 'colorectal cancer'/exp

#2. 'colon cancer'/exp

#3. 'rectum cancer'/exp

#4. #1 OR #2 OR #3

#5. #4 OR 'colonic neoplasm':ab,ti OR 'neoplasm, colonic':ab,ti OR 'neoplasms, colonic':ab,ti OR 'colon neoplasms':ab,ti OR 'colon neoplasm':ab,ti OR 'neoplasm, colon':ab,ti OR 'neoplasms, colon':ab,ti OR 'cancer of colon':ab,ti OR 'colon ancers':ab,ti OR 'cancer of the colon':ab,ti OR 'colonic cancer':ab,ti OR 'cancer, colonic':ab,ti OR 'cancers, colonic':ab,ti OR 'colonic cancers':ab,ti OR 'colon cancer':ab,ti OR 'cancer, colon':ab,ti OR 'cancers, colon':ab,ti OR 'colonic neoplasms':ab,ti OR 'rectalneoplasms':ab,ti OR 'neoplasm, rectal':ab,ti OR 'rectal neoplasm':ab,ti OR 'rectum neoplasms':ab,ti OR 'neoplasm, rectum':ab,ti OR 'rectum neoplasm':ab,ti OR 'rectal tumors':ab,ti OR 'rectal tumor':ab,ti OR 'tumor, rectal':ab,ti OR 'neoplasms, rectal':ab,ti OR 'cancer of rectum':ab,ti OR 'rectum cancers':ab,ti OR 'rectal cancer':ab,ti OR 'cancer, rectal':ab,ti OR 'rectal cancers':ab,ti OR 'rectum cancer':ab,ti OR 'cancer, rectum':ab,ti OR 'cancer of the rectum':ab,ti OR 'colorectal neoplasms':ab,ti OR 'colorectal neoplasm':ab,ti OR 'neoplasm, colorectal':ab,ti OR 'neoplasms, colorectal':ab,ti OR 'colorectal tumors':ab,ti OR 'colorectal tumor':ab,ti OR 'tumor, colorectal':ab,ti OR 'tumors, colorectal':ab,ti OR 'colorectal cancer':ab,ti OR 'cancer, colorectal':ab,ti OR 'cancers, colorectal':ab,ti OR 'colorectal cancers':ab,ti OR 'colorectal carcinoma':ab,ti OR 'carcinoma, colorectal':ab,ti OR 'carcinomas, colorectal':ab,ti OR 'colorectal carcinomas':ab,ti

#6. 'fatty acid'/exp

#7. #6 OR 'fatty acids':ab,ti OR 'fatty acid':ab,ti OR 'fatty acids, esterified':ab,ti OR 'esterified fatty acids':ab,ti OR 'esterified fatty acid':ab,ti OR 'acid, esterified fatty':ab,ti OR 'fatty acid, esterified':ab,ti OR 'fatty acids, saturated':ab,ti OR 'saturated fatty acids':ab,ti OR 'saturated fatty acid':ab,ti OR 'acid, saturated fatty':ab,ti OR 'fatty acid, saturated':ab,ti OR 'aliphatic acids':ab,ti OR 'aliphatic acid':ab,ti OR 'acid, aliphatic':ab,ti OR 'Linolenic acid':ab,ti OR 'Eicosapentaenoic acid':ab,ti OR 'Docosapentaenoic acid':ab,ti OR 'Docosahexaenoic acid':ab,ti OR 'Linoleic acid':ab,ti OR 'arachidonic acid':ab,ti OR 'fat':ab,ti

#8. #5 AND #7

Literature search strategy in Cochrane Library: 708 (publication till 2022 Feb):

#1 MeSH descriptor: [Colorectal Neoplasms] explode all trees

#2 MeSH descriptor: [Colonic Neoplasms] explode all trees

#3 MeSH descriptor: [Rectal Neoplasms] explode all trees

#4 #1 or #2 or #3

#5 #4 or ((Colonic Neoplasm):ti,ab,kw or (Neoplasm, Colonic):ti,ab,kw or (Neoplasms, Colonic):ti,ab,kw or (Colon Neoplasms):ti,ab,kw or (Colon Neoplasm):ti,ab,kw or (Neoplasm, Colon):ti,ab,kw or (Neoplasms, Colon):ti,ab,kw or (Cancer of Colon):ti,ab,kw or (Colon Cancers):ti,ab,kw or (Cancer of the Colon):ti,ab,kw or (Colonic Cancer):ti,ab,kw or (Cancer, Colonic):ti,ab,kw or (Cancers, Colonic):ti,ab,kw or (Colonic Cancers):ti,ab,kw or (Colon Cancer):ti,ab,kw or (Cancer, Colon):ti,ab,kw or (Cancers, Colon):ti,ab,kw or (Colonic Neoplasms):ti,ab,kw or (Neoplasm, Rectal):ti,ab,kw or (Rectal Neoplasms):ti,ab,kw or (Rectal Neoplasm):ti,ab,kw or (Rectum Neoplasms):ti,ab,kw or (Neoplasm, Rectum):ti,ab,kw or (Rectum Neoplasm):ti,ab,kw or (Rectal Tumors):ti,ab,kw or (Rectal Tumor):ti,ab,kw or (Tumor, Rectal):ti,ab,kw or (Neoplasms, Rectal):ti,ab,kw or (Cancer of Rectum):ti,ab,kw or (Rectum Cancers):ti,ab,kw or (Rectal Cancer):ti,ab,kw or (Cancer, Rectal):ti,ab,kw or (Rectal Cancers):ti,ab,kw or (Rectum Cancer):ti,ab,kw or (Cancer, Rectum):ti,ab,kw or (Cancer of the Rectum):ti,ab,kw or (Colorectal Neoplasms):ti,ab,kw or (Colorectal Neoplasm):ti,ab,kw or (Neoplasm, Colorectal):ti,ab,kw or (Neoplasms, Colorectal):ti,ab,kw or (Colorectal Tumors):ti,ab,kw or (Colorectal Tumor):ti,ab,kw or (Tumor, Colorectal):ti,ab,kw or (Tumors, Colorectal):ti,ab,kw or (Colorectal Cancer):ti,ab,kw or (Cancer, Colorectal):ti,ab,kw or (Cancers, Colorectal):ti,ab,kw or (Colorectal Cancers):ti,ab,kw or (Colorectal Carcinoma):ti,ab,kw or (Carcinoma, Colorectal):ti,ab,kw or (Carcinomas, Colorectal):ti,ab,kw or (Colorectal Carcinomas):ti,ab,kw )

#6 MeSH descriptor: [Fatty Acids] explode all trees

#7 #6 or ((Fatty Acids):ti,ab,kw or (Fatty Acid):ti,ab,kw or (Fatty Acids, Esterified):ti,ab,kw or (Esterified Fatty Acids):ti,ab,kw or (Esterified Fatty Acid):ti,ab,kw or (Acid, Esterified Fatty):ti,ab,kw or (Fatty Acid, Esterified):ti,ab,kw or (Fatty Acids, Saturated):ti,ab,kw or (Saturated Fatty Acids):ti,ab,kw or (Saturated Fatty Acid):ti,ab,kw or (Acid, Saturated Fatty):ti,ab,kw or (Fatty Acid, Saturated):ti,ab,kw or (Aliphatic Acids):ti,ab,kw or (Aliphatic Acid):ti,ab,kw or (Acid, Aliphatic):ti,ab,kw or (Linolenic acid):ti,ab,kw or (Eicosapentaenoic acid):ti,ab,kw or (Docosapentaenoic acid):ti,ab,kw or (Docosahexaenoic acid):ti,ab,kw or (Linoleic acid):ti,ab,kw or (arachidonic acid):ti,ab,kw or (fat):ti,ab,kw)

#8 #5 and #7

# Supplementary Figures and Tables

## Supplementary Figures

**
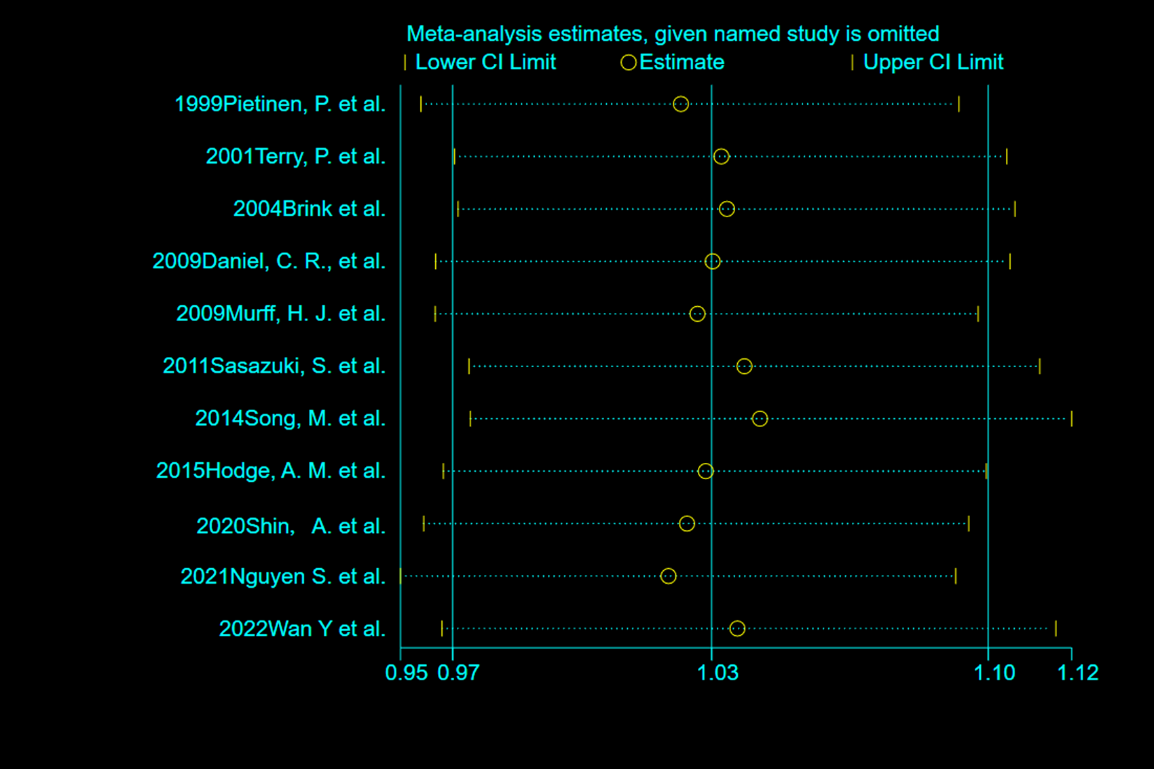
**

**Supplementary Figure 1.** Sensitivity analysis on dietary Alpha-linolenic Acid in relation to risk of colorectal cancer in which the pooled relative risk is re-estimated after omitting one study


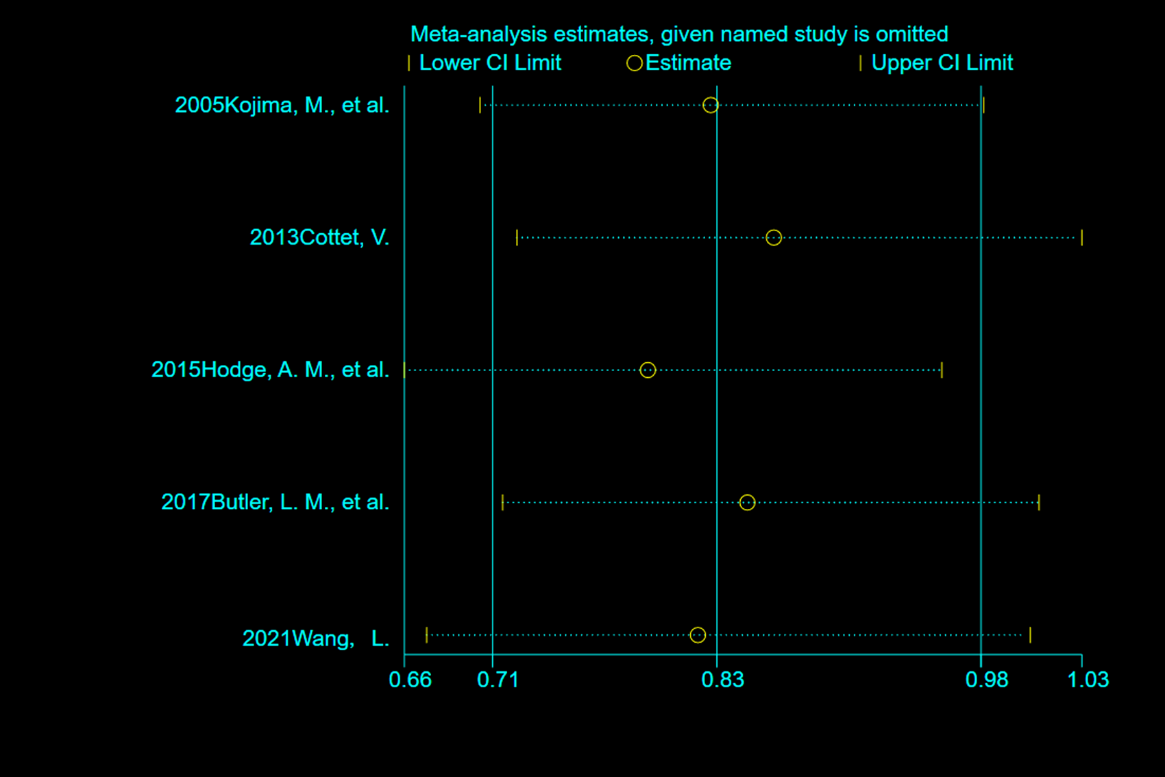


**Supplementary Figure 2.** Sensitivity analysis on biomarker Alpha-linolenic Acid in relation to risk of colorectal cancer in which the pooled relative risk is re-estimated after omitting one study


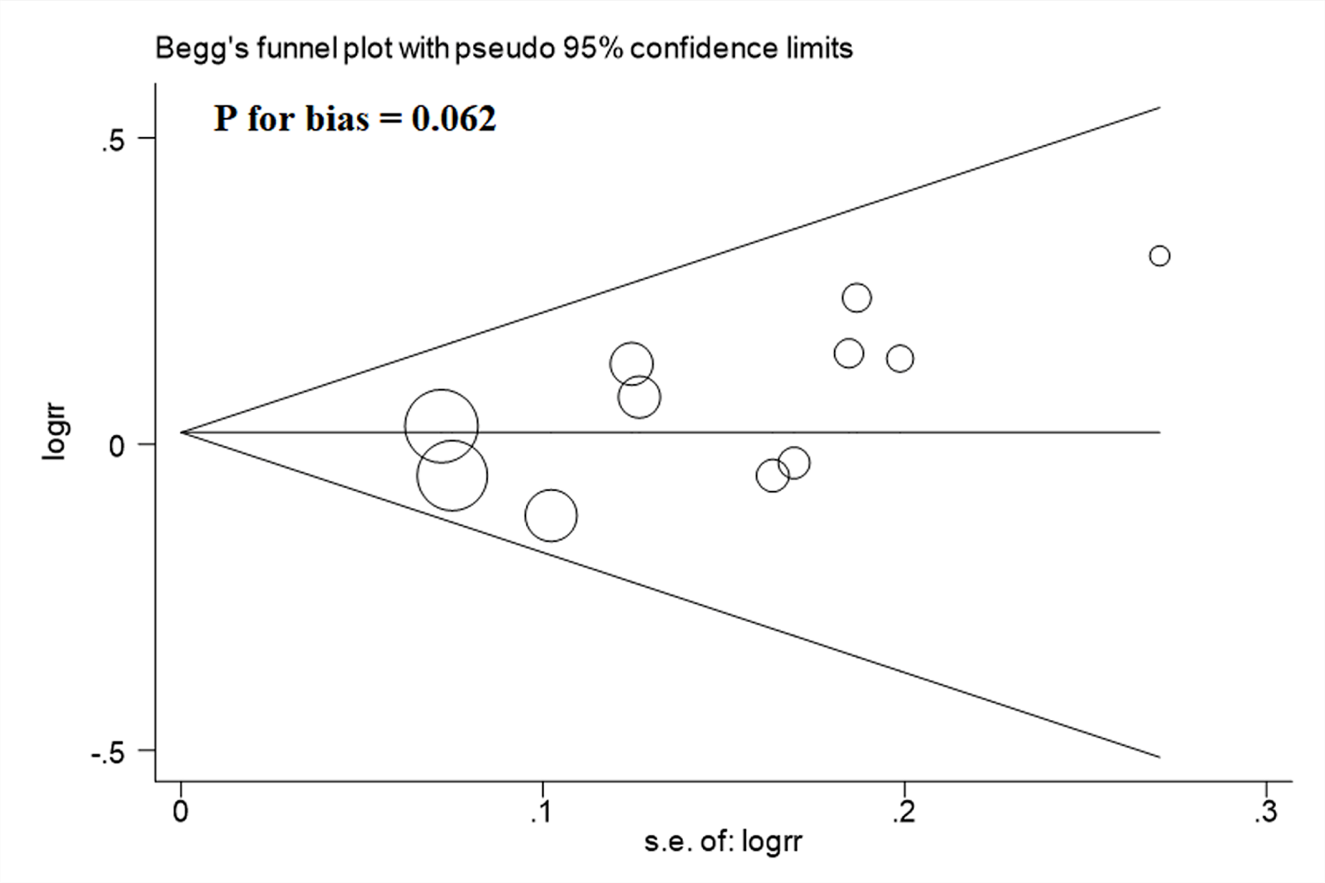


**Supplementary Figure 3.** Begg’s funnel plot on dietary Alpha-linolenic Acid in relation to risk of colorectal cancer


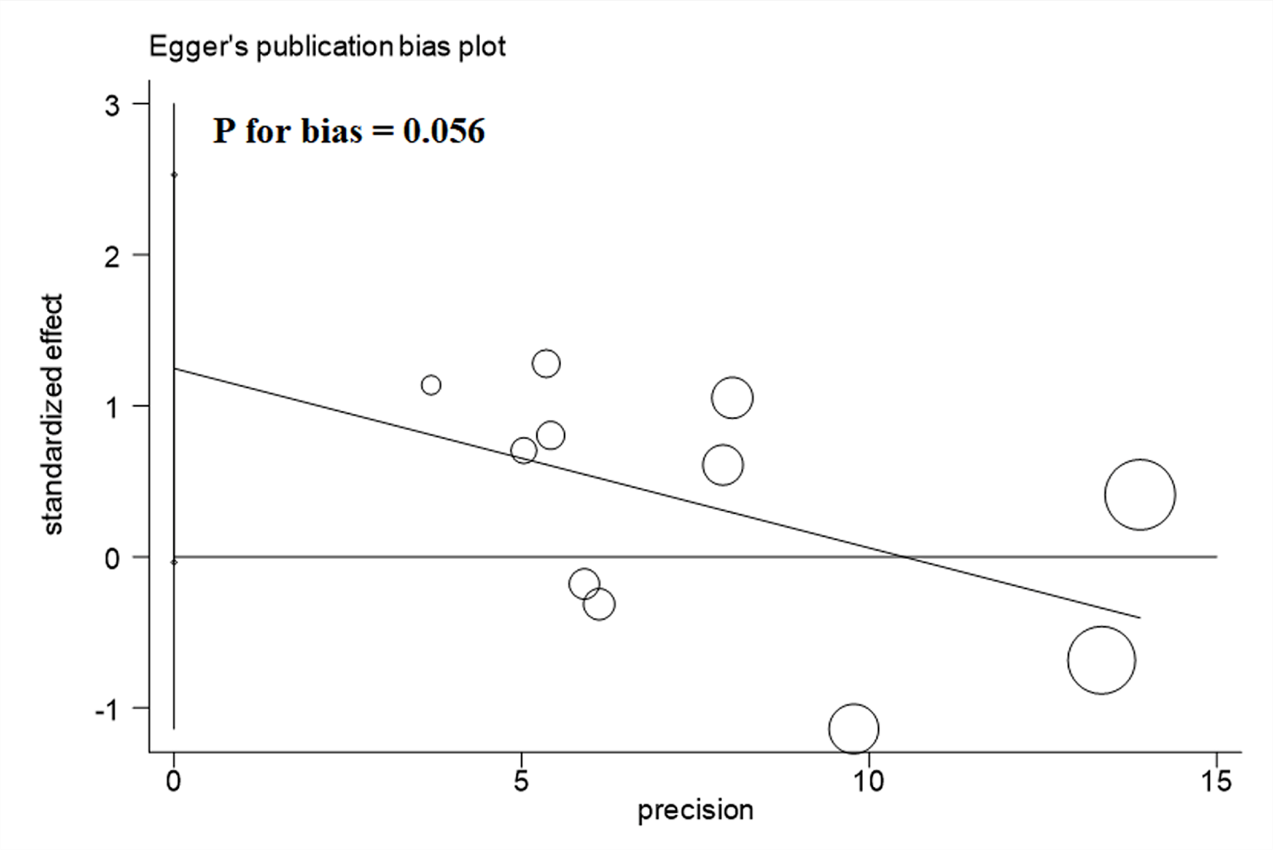


**Supplementary Figure 4.** Egger’s regress plot on dietary Alpha-linolenic Acid in relation to risk of colorectal cancer


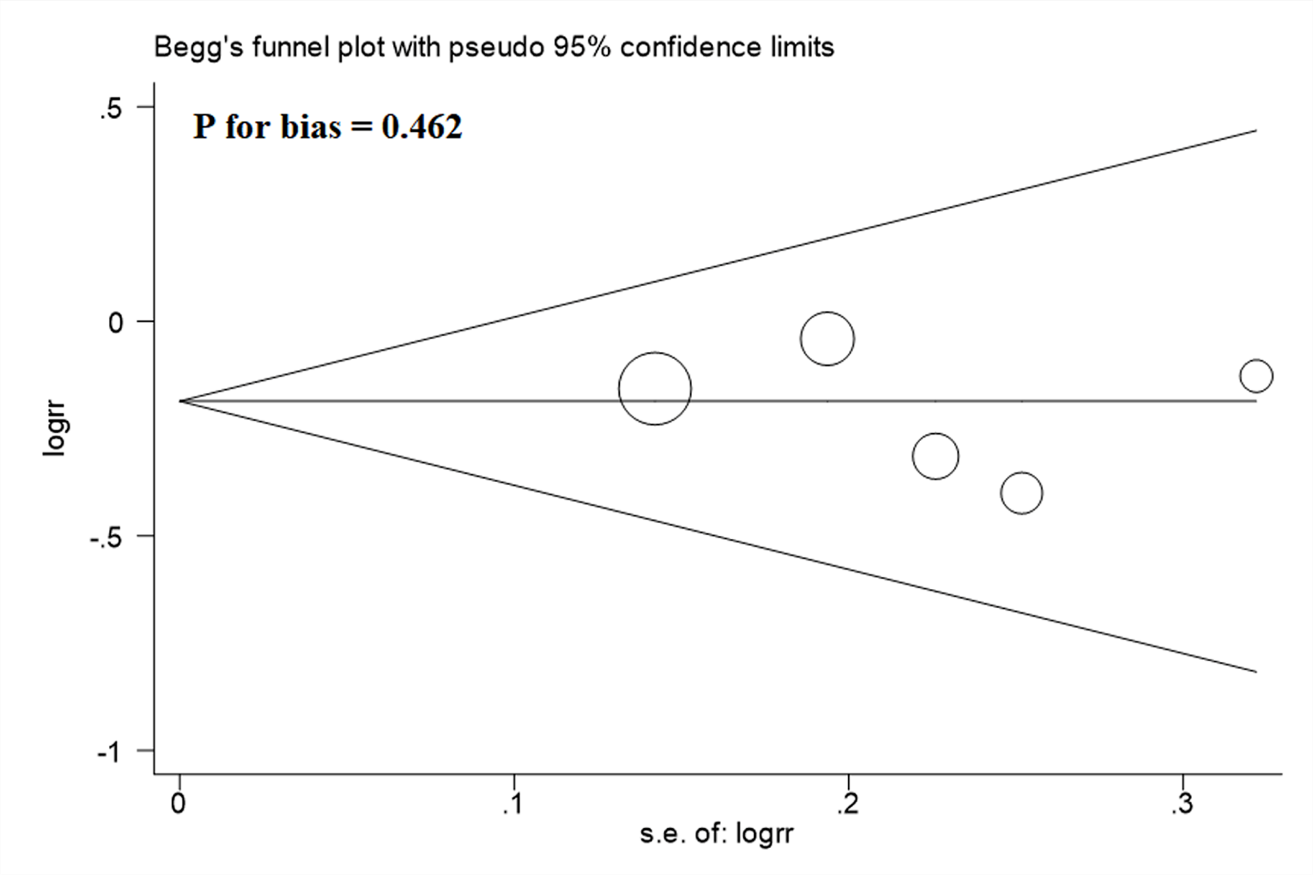


**Supplementary Figure 5.** Begg’s funnel plot on biomarkers of Alpha-linolenic Acid in relation to risk of colorectal cancer


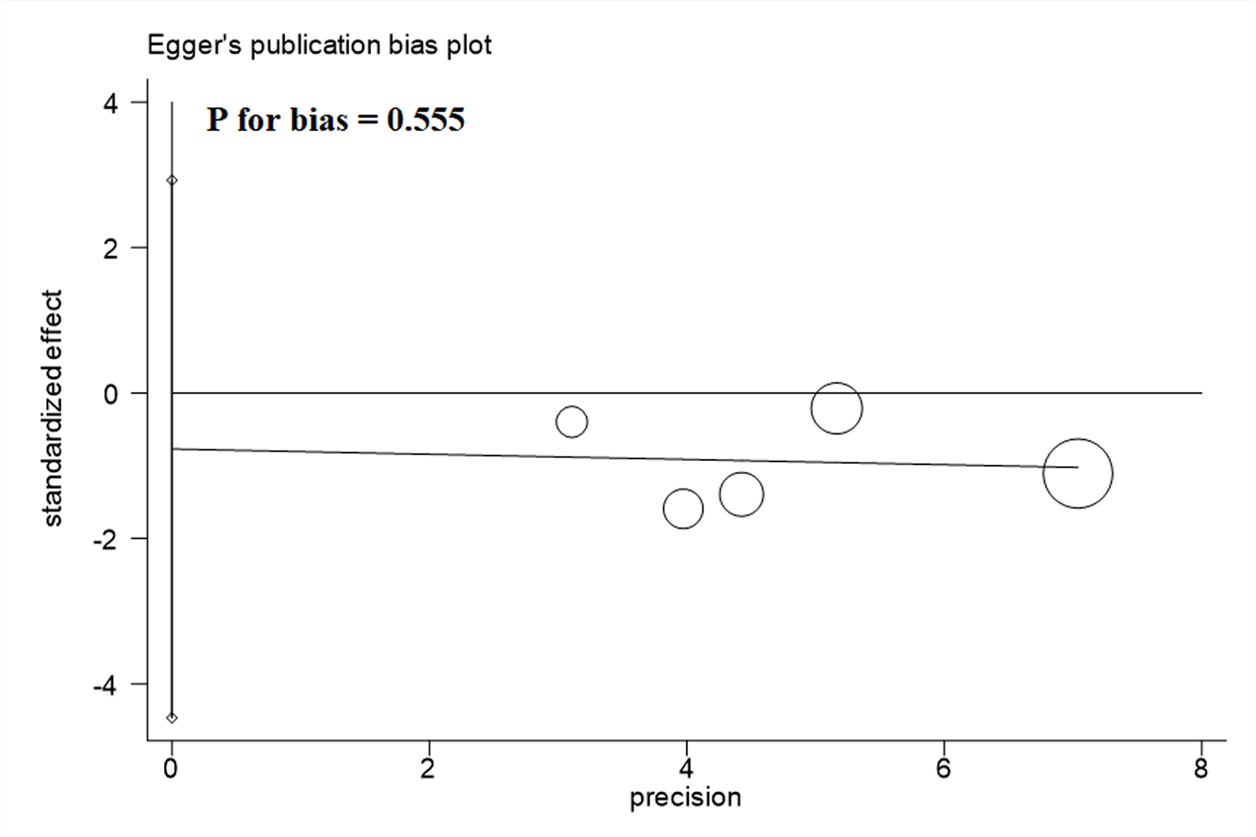


**Supplementary Figure 6.** Egger’s regress plot on biomarkers of Alpha-linolenic Acid in relation to risk of colorectal cancer


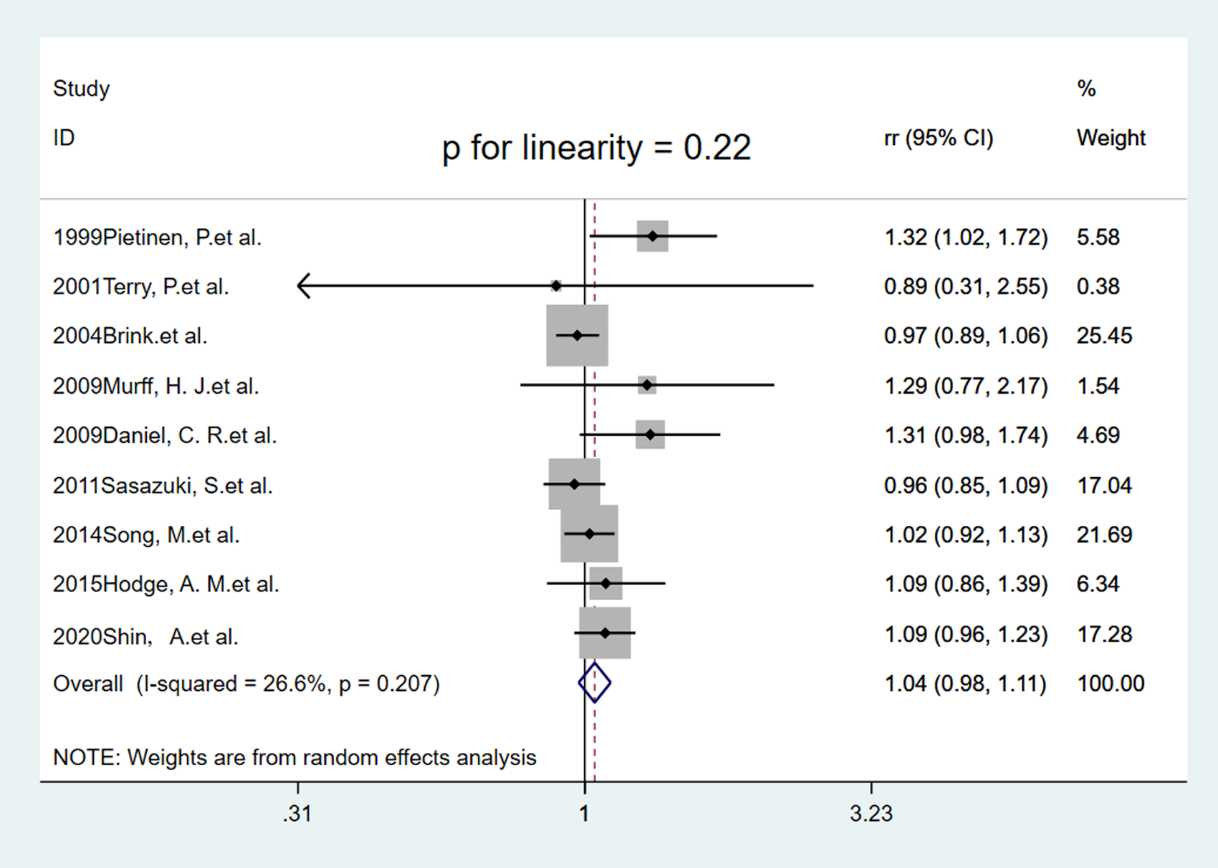


**Supplementary Figure 7.** Two-stage dose-response association of each 1.0-g/day increment of dietary Alpha-linolenic Acid with risk of colorectal cancer


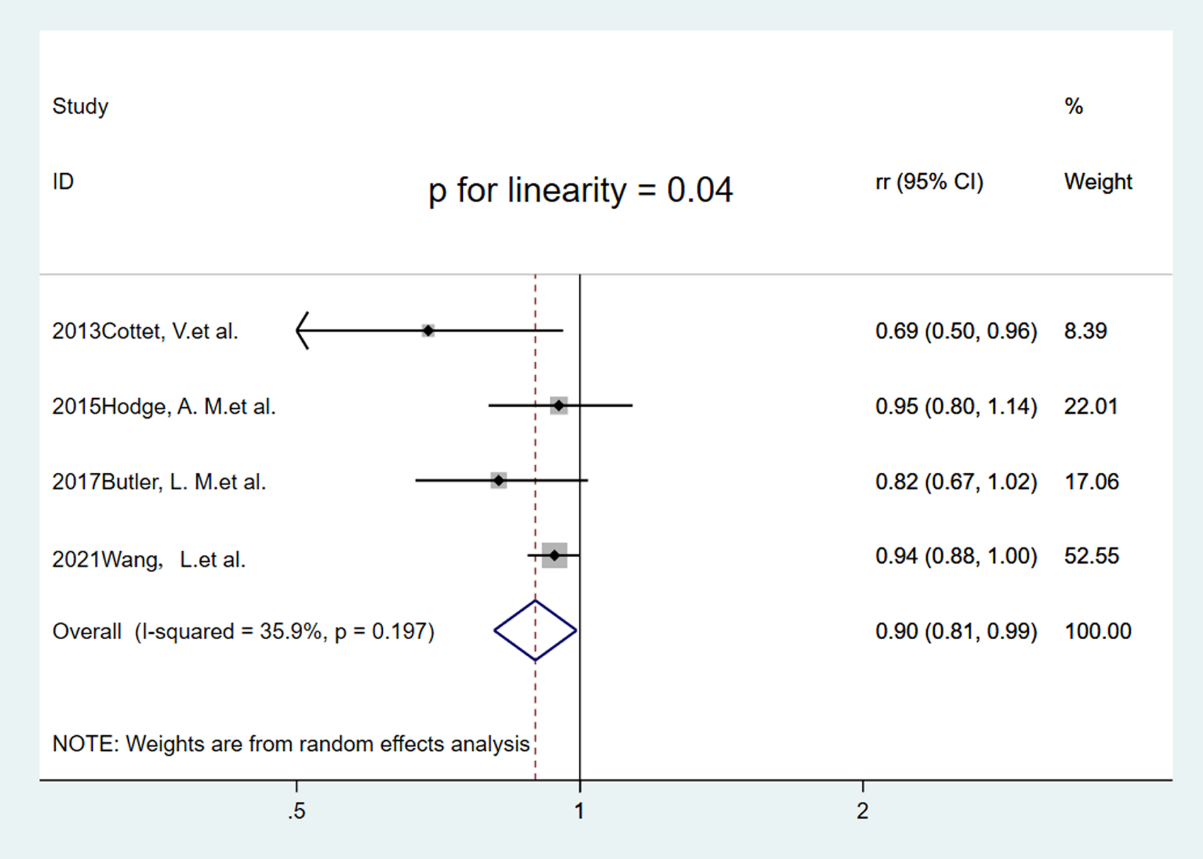


**Supplementary Figure 8.** Two-stage dose-response association of each 0.1% increment of circulating Alpha-linolenic Acid with risk of colorectal cancer

## Supplementary Tables

**Supplementary Table 1.** Reasons for exclusion of studies after full-text scrutiny

| **Reference title** | **Reasons for exclusion** |
| --- | --- |
| 1.Lipid chemical differences between human cancerous and adenomatous polypous tissues in the large intestine | selecting cancer patients as the control group |
| 2.Dietary fat, adipose tissue composition, and the development of carcinoma of the colon | designed as a retrospective study |
| 3.Dietary fat in relation to fatty acid composition of red cells and adipose tissue in colorectal cancer | designed as a retrospective study |
| 4.Relation of meat, fat, and fiber intake to the risk of colon cancer in a prospective study among women | not providing datas on ALA |
| 5.Arachidonic acid and docosahexaenoic acid are increased in human colorectal cancer | selecting cancer patients as the control group |
| 6.Effects of different doses of fish oil on rectal cell proliferation in patients with sporadic colonic adenomas | designed as a randomized controlled trial |
| 7.Sugar, meat, and fat intake, and non-dietary risk factors for colon cancer incidence in Iowa women (United States) | not providing datas on ALA |
| 8.Intake of fat, meat, and fiber in relation to risk of colon cancer in men | not providing datas on ALA |
| 9.A prospective cohort study on the relation between meat consumption and the risk of colon cancer | not providing datas on ALA |
| 10.Effects of n-3 PUFA on cell proliferation and vitamin E content in human subjects at high risk for colon cancer | designed as a randomized controlled trial |
| 11.Modulation of cytokine production in vivo by dietary essential fatty acids in patients with colorectal cancer | selecting cancer patients as the control group |
| 12.Missing anti-proliferative effect of fish oil on rectal epithelium in healthy volunteers consuming a high-fat diet: potential role of the n-3: n-6 fatty acid ratio | designed as a randomized controlled trial |
| 13.Effects of fish oil on fecal bacterial enzymes and steroid excretion in healthy volunteers: implications for colon cancer prevention | designed as a randomized controlled trial |
| 14.Changes of the mucosal n3 and n6 fatty acid status occur early in the colorectal adenoma-carcinoma sequence | designed as a retrospective study |
| 15.n-3 Fatty acids decrease colonic epithelial cell proliferation in high- risk bowel mucosa | designed as a randomized controlled trial |
| 16.Adipose fatty acids and cancers of the breast, prostate and colon: An ecological study | not providing datas on ALA |
| 17.Dietary fats and colon cancer: assessment of risk associated with specific fatty acids | not providing datas on ALA |
| 18.Abnormalities in plasma and red blood cell fatty acid profiles of patients with colorectal cancer | designed as a retrospective study |
| 19.Serum n-3 fatty acids, fish consumption and cancer mortality in six Japanese populations in Japan and Brazil | not evaluating CRC incidence as an end point |
| 20.Dietary fat, cholesterol and colorectal cancer in a prospective study | not providing datas on ALA |
| 21.An ecological study of dietary fat intake and mortality rates from breast cancer and colorectal cancer in Taiwanese women | not evaluating CRC incidence as an end point |
| 22.Tissue levels of fish fatty acids and risk of colorectal adenomas: a case-control study (Netherlands) | not providing datas on ALA |
| 23.Assessment of risk associated with specific fatty acids and colorectal cancer among French-Canadians in Montreal: a case-control study | designed as a retrospective study |
| 24.Fish, long-chain n-3 polyunsaturated fatty acids, and risk of colorectal cancer in middle-aged Japanese: the JPHC study | not providing datas on ALA |
| 25.Interaction between cyclooxygenose-2 gene polymorphism and dietary n-6 polyunsaturated fatty acids on colon cancer risk: The Singapore Chinese Health Study | not providing datas on ALA |
| 26.Dietary fat and fatty acids and risk of colorectal cancer in women | not providing datas on ALA |
| 27.n-3 polyunsaturated fatty acids and colon cancer prevention | no relevant study |
| 28.[Dietary fatty acids and colorectal and prostate cancers: epidemiological studies] | no relevant study |
| 29.High-fat dairy food and conjugated linoleic acid intakes in relation to colorectal cancer incidence in the Swedish Mammography Cohort | no relevant study |
| 30.Meat, milk, saturated fatty acids, the Pro12Ala and C161T polymorphisms of the PPARγ gene and colorectal cancer risk in Japanese | subgroup data for the included studies |
| 31.Risk of colorectal cancer is linked to erythrocyte compositions of fatty acids as biomarkers for dietary intakes of fish, fat, and fatty acids | designed as a retrospective study |
| 32.The relationship between the consumption of meat, fat, and coffee and the risk of colon cancer: a prospective study in Japan | not providing datas on ALA |
| 33.Blood levels of long-chain polyunsaturated fatty acids, aspirin, and the risk of colorectal cancer | not providing datas on ALA |
| 34.Meat, fish and fat intake in relation to subsite-specific risk of colorectal cancer: The Fukuoka Colorectal Cancer Study | not providing datas on ALA |
| 35.Dietary fatty acids and colorectal cancer: a case-control study | designed as a retrospective study |
| 36.Serum concentrations of fatty acids and colorectal adenoma risk: a case study in Japan | designed as a retrospective study |
| 37.A 22-year prospective study of fish, n-3 fatty acid intake, and colorectal cancer risk in men | not providing datas on ALA |
| 38.Prospective evaluation of trans-fatty acid intake and colorectal cancer risk in the Iowa Women's Health Study | no relevant study |
| 39.Effects of ω-3 polyunsaturated fatty acids on colon cancer | no relevant study |
| 40.Opposing associations of serum n-3 and n-6 polyunsaturated fatty acids with colorectal adenoma risk: an endoscopy-based case-control study | designed as a retrospective study |
| 41.Marine n-3 and saturated fatty acids in relation to risk of colorectal cancer in Singapore Chinese: a prospective study | not providing datas on ALA |
| 42.Animal origin foods and colorectal cancer risk: a report from the Shanghai Women's Health Study | not providing datas on ALA |
| 43.Polyunsaturated fatty acids, DNA repair single nucleotide polymorphisms and colorectal cancer in the Singapore Chinese Health Study | no relevant study |
| 44.Intake of dietary fats and colorectal cancer risk: Prospective findings from the UK Dietary Cohort Consortium | not providing datas on ALA |
| 45.Associations of red meat, fat, and protein intake with distal colorectal cancer risk | not providing datas on ALA |
| 46.Vitamins, minerals, essential fatty acids and colorectal cancer risk in the United Kingdom Dietary Cohort Consortium | not providing datas on ALA |
| 47.A randomized controlled trial of eicosapentaenoic acid and/or aspirin for colorectal adenoma prevention during colonoscopic surveillance in the NHS Bowel Cancer Screening Programme (The seAFOod Polyp Prevention Trial): study protocol for a randomized controlled trial | no relevant study |
| 48.Abnormalities in fatty acids in plasma, erythrocytes and adipose tissue in Japanese patients with colorectal cancer | designed as a retrospective study |
| 49.[Clinicopathological significance of abnormal metabolism of polyunsaturated fatty acids in colorectal cancer tissue] | selecting cancer patients as the control group |
| 50.Characteristics of fatty acid distribution is associated with colorectal cancer prognosis | selecting cancer patients as the control group |
| 51.Dietary fat, fatty acid intakes and colorectal cancer risk in Chinese adults: a case-control study | designed as a retrospective study |
| 52.Erythrocyte membrane fatty acids profile in colorectal cancer patients: a preliminary study | designed as a retrospective study |
| 53.Targeted analysis of progressive metabolic perturbations in colorectal cancer in colorectal adenoma: Potential for a serum metabolomics-based colorectal cancer screening test | no relevant study |
| 54.Adipose tissue fatty acid composition and colon cancer: a case-control study | designed as a retrospective study |
| 55.Long-chain omega-3 polyunsaturated fatty acid intake and risk of colorectal cancer | not providing datas on ALA |
| 56.Dietary Fiber Intake Modifies the Positive Association between n-3 PUFA Intake and Colorectal Cancer Risk in a Caucasian Population | not providing datas on ALA |
| 57.The relationship between dietary ω-3 and ω-6 intake and colorectal cancer | not providing datas on ALA |
| 58.Erythrocyte membrane phospholipid fatty acid concentrations and colorectal adenomas: A case control in tennessee | not providing datas on ALA |
| 59.Associations of colorectal cancer incidence with nutrient and food group intakes in korean adults: a case-control study | not providing datas on ALA |
| 60.Fatty acid composition of adipose tissue and colorectal cancer: a case-control study | designed as a retrospective study |
| 61.The Interaction between Dietary Fiber and Fat and Risk of Colorectal Cancer in the Women's Health Initiative | not providing datas on ALA |
| 62.Marine ω-3 polyunsaturated fatty acids and risk of colorectal cancer according to microsatellite instability | no relevant study |
| 63.Dietary n-3/long-chain n-3 polyunsaturated fatty acids for prevention of sporadic colorectal tumors: a randomized controlled trial in polypectomized participants | designed as a randomized controlled trial |
| 64.Differences of polyunsaturated fatty acid in patients with colorectal cancer and healthy people | designed as a retrospective study |
| 65.Expression profile of polyunsaturated fatty acids in colorectal cancer | selecting cancer patients as the control group |
| 66.Role of serum polyunsaturated fatty acids in the development of colorectal cancer | designed as a retrospective study |
| 67.Dietary polyunsaturated fatty acids intake modifies the positive association between serum total cholesterol and colorectal cancer risk: the Rotterdam Study | not providing datas on ALA |
| 68.Altered Saturated and Monounsaturated Plasma Phospholipid Fatty Acid Profiles in Adult Males with Colon Adenomas | no relevant study |
| 69.Marine ω-3 Polyunsaturated Fatty Acid Intake and Risk of Colorectal Cancer Characterized by Tumor-Infiltrating T Cells | subgroup data for the included studies |
| 70.Marine omega-3 polyunsaturated fatty acid intake and survival after colorectal cancer diagnosis | not evaluating CRC incidence as an end point |
| 71.Measurement of red blood cell eicosapentaenoic acid (EPA) levels in a randomised trial of EPA in patients with colorectal cancer liver metastases | designed as a randomized controlled trial |
| 72.Serum Unsaturated Free Fatty Acids: A Potential Biomarker Panel for Early-Stage Detection of Colorectal Cancer | not using RR(HR) as outcomes |
| 73.Association between dietary fat intake and colorectal adenoma in korean adults: A cross-sectional study | not providing datas on ALA |
| 74.Pro-inflammatory fatty acid profile and colorectal cancer risk: A Mendelian randomisation analysis | no relevant study |
| 75.Long-chain ω-6 plasma phospholipid polyunsaturated fatty acids and association with colon adenomas in adult men: A cross-sectional study | not providing datas on ALA |
| 76.PUFA levels in erythrocyte membrane phospholipids are differentially associated with colorectal adenoma risk | not providing datas on ALA |
| 77.Marine omega-3 polyunsaturated fatty acid and colorectal cancer prevention and treatment | not evaluating CRC incidence as an end point |
| 78.Long-chain omega-3 fatty acid and fish intake after colon cancer diagnosis and disease-free, recurrence-free, and overall survival in CALGB 89803 (Alliance) | no relevant study |
| 79.Distinct differences in serum eicosanoids in healthy, enteritis and colorectal cancer individuals | no relevant study |
| 80.Serum polyunsaturated fatty acid metabolites as useful tool for screening potential biomarker of colorectal cancer | no relevant study |
| 81.Altered red blood cell membrane fatty acid profile in cancer patients | not evaluating CRC incidence as an end point |
| 82.LIFESTYLE FACTORS AND RISK OF SERRATED LESION AND CONVENTIONAL ADENOMA: A PROSPECTIVE STUDY OF THREE US COHORTS | not providing datas on ALA |
| 83.Eicosapentaenoic acid (EPA) and/or aspirin for prevention of colorectal adenomas (the seafood polyp prevention trial): a multicentre double-blind, placebo-controlled, randomised 2X2 factorial phase 3 trial | designed as a randomized controlled trial |
| 84.Erythrocyte membrane fatty acids as the potential biomarkers for detection of early-stage and progression of colorectal cancer | no relevant study |
| 85.Associations of dietary fat with risk of early neoplasia in the proximal colon in a population-based case-control study | not providing datas on ALA |
| 86.Pilot study: high dose fish oil in colorectal cancer (CRC) prevention in patients with lynch syndrome | no relevant study |
| 87.No risk reduction of colorectal adenoma with aspirin or eicosapentaenoic acid | designed as a randomized controlled trial |
| 88.Arachidonic acid and colorectal adenoma risk: a Mendelian randomization study | no relevant study |
| 89.Perturbation of fatty acids of erythrocyte membranes and blood serum in patients with colorectal cancer: new opportunities for diagnostics | no relevant study |
| 90.Electrical and viscoelastic parameters of erythrocytes in combination with the fatty acid profile of their membranes and serum as potential biom arkers for the diagnosis of colorectal cancer | no relevant study |
| 91.Saturated, mono- and polyunsaturated fatty acid intake and cancer risk: results from the French prospective cohort NutriNet-Santé | not evaluating CRC incidence as an end point |
| 92.Marine omega-3 fatty acid supplementation and risk of colorectal adenomas and serrated polyps: a randomized placebo-controlled trial | designed as a randomized controlled trial |
| 93.Consumption of Fish and Long-chain n-3 Polyunsaturated Fatty Acids Is Associated With Reduced Risk of Colorectal Cancer in a Large European Cohort | not providing datas on ALA |
| 94.Mendelian Randomization of Circulating Polyunsaturated Fatty Acids and Colorectal Cancer Risk | designed as a retrospective study |
| 95.P-3 Features of metabolic profiles of blood serum and erythrocyte membranes associated with metastasis in colorectal cancer | no relevant study |
| 96.Blood serum and erythrocyte lipidomic profiling revealed diagnostic biomarkers in patients with early stages of crc and adenomatous polyps | no relevant study |
| 97.A conjunctive lipidomic approach reveals plasma ethanolamine plasmalogens and fatty acids as early diagnostic biomarkers for colorectal cancer patients | no relevant study |
| 98.Dietary intake and plasma phospholipid concentrations of saturated, monounsaturated and trans fatty acids and colorectal cancer risk in the European Prospective Investigation into Cancer and Nutrition cohort | no relevant study |
| 99.P-15 Diagnostic potential of erythrocyte and serum fatty acids in spotting adenomatous polyps and identifying the early stages of colorectal cancer depending on tumor localization | Meeting abstract |
| 100.Genetically predicted plasma phospholipid arachidonic acid concentrations and 10 site-specific cancers in UK biobank and genetic consortia participants: A mendelian randomization study | no relevant study |
| 101.N-3 Long Chain Fatty Acids Supplementation, Fatty Acids Desaturase Activity, and Colorectal Cancer Risk: a Randomized Controlled Trial | designed as a randomized controlled trial |
| 102.Free Fatty Acid is a Promising Biomarker in Triage Screening for Patients with Colorectal Cancer: A Case-Control Study | no relevant study |
| 103.Combined use of findings of levels of fatty acids in erythrocyte membranes and blood serum as well as electric, viscoelastic parameters of erythrocytes increases accuracy of diagnosis of pre-cancer and early colorectal cancer | not using RR(HR) as outcomes |
| 104.Dietary Risk Factors and Odds of Colorectal Adenoma in Malaysia: A Case Control Study | not providing datas on ALA |

**Supplementary Table 2.** Moose checklist of present meta-analysis

| Criteria | Comments of how the criteria were handled in the meta-analysis | Reported on page # |
| --- | --- | --- |
|  | Reporting of background should include |  |
| Problem definition | Colorectal cancer (CRC) is the fourth most deadly cancer worldwide. Alpha-linolenic acid (ALA, 18:3n-3), as a plant-based member of n-3 PUFAs, can be derived from vegetable oils. Most observational studies of ALA have assessed dietary intake by using food frequency questionnaires (FFQs) or weighed food records, which may have led to dietary measurement errors or bias. Biomarkers of ALA provide objective measures that reﬂect both dietary consumption and relevant biologic processes. So far, the relationships between ALA biomarker and CRC risk remain unclear. | 1 & 2 |
| Hypothesis statement | Dietary intake and biomarkers of ALA was inversely associated with incidence of CRC | 2 |
| Description of study outcomes | So far, the relationships between ALA intake and CRC risk remain unclear, as various prospective cohorts reported inconsistent results. | 2 |
| Type of exposure or intervention used | Dietary intake and biomarker levels of ALA | 2 |
| Type of study designs used | Systematic review and meta-analysis. | 2 |
| Study population | Adults of any age across different countries | 3 |
|  | Reporting of search strategy should include |  |
| Qualifications of searchers(eg. librarians and investigators) | Two trained reviewers are indicated in the author list. Discrepancies unsolved by discussion during the course of study identification consulted to a third reviewer. | 3 |
| Search strategy, including time period included in the synthesis and keywords | Using a method of the key works combined with medical subject headings, and the full details were presented in supplementary data (Supplementary Materials). | 2 |
| Databases and registries searched | PubMed, EMBASE and Cochrane Library database were searched, and we also check the reference lists to identify studies that might have been missed | 2 |
| Search software used, name and version, including special features | We did not employ search software. EndNote was used to merge retrieved citations and eliminate duplications | 2 |
| Use of hand searching | We hand-searched bibliographies of retrieved papers, and check the reference lists from systematic review to identify studies that might have been missed. | 2 |
| List of citations located and those excluded, including justifications | The all steps and details of the literature search process are outlined in the flow chart (Figure 1). | 4 |
| Method of addressing articles published in languages other than English | Our search was restricted to human studies, and studies published in English. | 4 |
| Method of handling abstracts and unpublished studies | Abstract, unpublished studies and duplicated study were excluded. | 4 |
| Description of any contact with authors | We did not contact authors for the detailed information of primary studies and unpublished studies. | 2 |
|  | Reporting of methods should include |  |
| Description of relevance or appropriateness of studies assembled for assessing the hypothesis to be tested | Detailed inclusion criteria were described in the methods section. | 3 |
| Rationale for the selection and coding of data | Data extracted from each of the studies were relevant to the population characteristics, study design, exposure, outcome, and adjusted confounding factors as covariates. | 3 |
| Assessment of confounding | Restricted the analysis to multiple covariates adjusted estimates. To provide a consistent approach to meta-analysis, the RR was transformed to involve comparisons between the top and the bottom quartiles of the population baseline ALA in dietary intake and proportions of biospecimens. A sensitivity analysis was conducted by eliminating included studies one by one. Publication bias was quantitatively examined by Begg’s test and Egger’s regression test. | 3 |
| Assessment of study quality, including blinding of quality assessors; stratification or regression on possible predictors of study results | We valuated study quality and risk of bias by using the Newcastle-Ottawa scale. Subgroup analyses and meta-regression analyses were conducted to identify the sources of living region, baseline age, gender, median duration of follow-up, cancer location, quality scores, study design, biomarker types and multiple adjustments | 3 |
| Assessment of heterogeneity | Heterogeneity of the studies were explored using Cochrane’s Q test of heterogeneity and I^2^ statistic that provides the relative amount of variance of the summary effect due to the between-study heterogeneity. | 3 |
| Description of statistical methods in sufficient detail to be replicated | Description of methods of meta-analyses for the top quartiles compared with the bottom, dose-response meta-analysis, subgroup analysis, sensitivity analyses and assessment of publication bias are detailed in the methods. | 3 & 4 |
| Provision of appropriate tables and graphics | Yes | 13 - 16 |
|  | Reporting of results should include |  |
| Graph summarizing individual study estimates and overall estimate | See meta-analysis for the top tertiles vs. the bottom (Figure 2 & 3) and dose-response trend (Figure 4).  All details in Sup. Figure 1-14. | 5 |
| Table giving descriptive information for each study included | See characteristics of the included studies (Table 1) | 13 - 16 |
| Results of sensitivity testing | See results of sensitivity analysis and subgroup analysis. (Sup. Figure S1 and S2; Table S4) | 5 |
| Indication of statistical uncertainty of findings | 95% confidence intervals were presented with all summary estimates, I2 values, results of sensitivity analyses and publication analysis.(Sup. Figure S1 - S8) | 5 |
|  | Reporting of discussion should include |  |
| Quantitative assessment of bias | Q test and I2 statistic indicated no heterogeneity in strengths of the relationship. Evaluation of results form stratified analyses. | 6-8 |
| Justification for exclusion | We performed sensitivity analysis omitting the study to reduce the influence of potential selective bias on the overall estimate, in view of probable selection bias. | 8 |
| Assessment of quality of included studies | We discussed the results of the sensitivity analyses, and potential reasons for the observed heterogeneity. | 8 |
|  | Reporting of conclusions should include |  |
| Consideration of alternative explanations for observed results | We found that biomarkers of ALA were inversely associated with the incident CRC, and each 0.1% increase in circulating levels of ALA was associated with 10% reduction in CRC risk. | 8 |
| Generalization of the conclusions | The present meta-analysis highlights that Encouraging the consumption of food rich in ALA to improve its levels in blood may potentially decrease the risk of CRC | 8 |
| Guidelines for future research | Well-designed and large-scale cohorts with biomarkers are still needed for better reconfirming the impacts of ALA intake in primary prevention of CRC. | 8 |
| Disclosure of funding source | This work is supported by the Ph. D. Programs Foundation of Wenzhou Medical University of Zhejiang Province, China (89217015), and by 2017 Chinese Nutrition Society (CNS) Nutrition Research Foundation—DSM Research Fund (95017008). | 8 |

**Supplementary Table 3.** Quality assessment of included prospective cohort studies by Newcastle-Ottawa scale (stars)

| **Study design** | **Selection(☆☆☆☆)** | | **Comparability(☆☆)** | **Exposure or Outcome (☆☆☆)** | | **Stars** | **Quality scores** |  |
| --- | --- | --- | --- | --- | --- | --- | --- | --- |
| **Prospective Cohort** | **Representativeness of the exposed cohort? ☆**  **Selection of the non exposed cohort? ☆**  **Evaluating exposure? ☆**  **Outcomes of interest were not present at study start? ☆** | | **1) Study controls for the most important factor? ☆**  **2) Study controls for any additional factors? ☆** | **How to ascertain outcome? ☆**  **a) Independent blindness**  **b) record linkage**  **Follow-up till outcomes happened? ☆**  **Adequacy of follow up? ☆** | | **☆☆☆☆☆☆☆☆☆ (9)** | **High quality: 8-9stars,**  **Moderate quality:**  **6-7stars,**  **Low quality:1-5 stars** |  |
| **Nested case-control/**  **Case Cohort** | **1) Adequate case definition? ☆**  **2) Representativeness of the cases? ☆**  **3) Community controls? ☆**  **4) Controls have no history of endpoint disease? ☆** | | **1) Study controls for the most important factor? ☆**  **2) Study controls for any additional factors? ☆** | **How to ascertain exposure?☆**  **a) By secure record**  **b)Structured interview where blind to case/control status**  **Same method of ascertainment for both? ☆**  **Same response rate for both? ☆** | |  |  |  |
| **Included Prospective Cohort** | | | | | | | |  |
| **1999 Pietinen, P. et al. (America)** | **1) ☆, 2) ☆, 3) ☆, 4) ☆** | | **1) ☆, 2) ☆** | **1) ☆, 2) ☆, 3) ☆** | | **☆☆☆☆☆☆☆☆☆(9)** | **High** |  |
| **2001Terry, P. et al. (Europe)** | **1) ☆, 2) ☆, 3) ☆, 4) ☆** | | **1) ☆, 2) ☆** | **1) ☆, 2) ☆,**  **3) ×: no description** | | **☆☆☆☆☆☆☆☆(8)** | **High** |  |
| **2009Daniel, C. R.et al. (America)** | **1) ☆, 2) ☆, 3) ☆, 4) ☆** | | **1) ☆, 2) ☆** | **1) ☆, 2) ☆, 3) ☆** | | **☆☆☆☆☆☆☆☆☆(9)** | **High** |  |
| **2009Murff, H. J.et al. (America)** | **1) ☆, 2) ☆, 3) ☆, 4) ☆** | | **1) ☆, 2) ☆** | **1) ☆, 2) ☆, 3) ☆** | | **☆☆☆☆☆☆☆☆☆(9)** | **High** |  |
| **2011Sasazuki, S.et al. (Asia)** | **1) ☆, 2) ☆, 3) ☆, 4) ☆** | | **1) ☆, 2) ☆** | **1) ☆, 2) ☆, 3) ☆** | | **☆☆☆☆☆☆☆☆☆(9)** | **High** |  |
| **2014Song, M.et al. (America)** | **×: selected group of nurses**  **2) ☆, 3) ☆, 4) ☆** | | **1) ☆, 2) ☆** | **1) ☆, 2) ☆,**  **3) ×: follow up rate <80%** | | **☆☆☆☆☆☆☆☆(7)** | **Medium** |  |
| **2020Shin, A. et al. (Europe)** | **1) ☆, 2) ☆, 3) ☆, 4) ☆** | | **1) ☆, 2) ☆** | **1) ☆, 2) ☆, 3) ☆** | | **☆☆☆☆☆☆☆☆☆(9)** | **High** |  |
| **2021Nguyen S. et al. (Asia)** | **1) ☆, 2) ☆, 3) ☆, 4) ☆** | | **1) ☆, 2) ☆** | **1) ☆, 2) ☆, 3) ☆** | | **☆☆☆☆☆☆☆☆☆(9)** | **High** |  |
| **2022Wan Y. et al. (America)** | 1. **×: selected group of nurses**   **2) ☆, 3) ☆, 4) ☆** | | **1) ☆, 2) ☆** | **1) ☆, 2) ☆,**  **3) ×: follow up rate <80%** | | **☆☆☆☆☆☆☆☆(7)** | **Medium** |  |
| **Included Nested Case-Control Studies/Case Cohort** | | | | | | | |  |
| **Wennberg et al, 2007 (Europe)** | **☆, 2) ☆, 3) ☆ ,**  **4) ×: no mention** | **1) ☆, 2) ☆** | | | **1) ☆, 2) ☆, 3) ☆** | **☆☆☆☆☆☆☆☆** | **High** | |
| **2004Brink. et al. (Europe)** | **☆, 2) ☆, 3) ☆ , 4) ☆** | **1) ☆, 2) ☆** | | | **1) ☆, 2) ☆, 3) ☆** | **☆☆☆☆☆☆☆☆☆(9)** | **High** | |
| **2005Kojima, M.et al. (Asia)** | **☆, 2) ☆, 3) ☆ , 4) ☆** | **1) ☆, 2) ☆** | | | **1) ☆, 2) ☆, 3) ☆** | **☆☆☆☆☆☆☆☆☆(9)** | **High** | |
| **2013Cottet, V.et al. (Europe)** | **☆, 2) ×: selected group of volunteers, 3) ☆ , 4) ☆** | **1) ☆, 2) ☆** | | | **1) ☆, 2) ☆, 3) ☆** | **☆☆☆☆☆☆☆☆☆(8)** | **High** | |
| **2015Hodge, A. M.et al. (Europe)** | **☆, 2) ☆, 3) ☆ , 4) ☆** | **☆,**  **2) ×: lost control for age** | | | **1) ☆, 2) ☆, 3) ☆** | **☆☆☆☆☆☆☆☆☆(8)** | **High** | |
| **2017Butler, L. M.et al. (Asia)** | **☆, 2) ☆, 3) ☆ , 4) ☆** | **1) ☆, 2) ☆** | | | **1) ☆, 2) ☆, 3) ☆** | **☆☆☆☆☆☆☆☆☆(9)** | **High** | |
| **2021Wang, L.et al. (America)** | **☆, 2) ☆, 3) ☆ , 4) ☆** | **1) ☆, 2) ☆** | | | **1) ☆, 2) ☆, 3) ☆** | **☆☆☆☆☆☆☆☆☆(9)** | **High** | |

**Supplementary Table 4.** Subgroup analyses for dietary Alpha-linolenic Acid in the top quartile compared with the bottom

| **Factors stratified** | **Dietary intake** | | | | |  |
| --- | --- | --- | --- | --- | --- | --- |
|  | **N** | **RR(95% CI)** | **Heterogeneity** | | **P**2 |  |
|  |  |  | **I**2 **(%)** | **P**1 |  |  |
| Overall analysis | 11 | 1.02 (0.95, 1.09) | 0.00 | 0.69 |  |  |
| Area |  |  |  |  | 0.65 |  |
| America | 4 | 1.02 (0.93, 1.11) | 0.00 | 0.49 |  |  |
| Europe | 4 | 1.04 (0.90, 1.21) | 0.00 | 0.82 |  |  |
| Asia | 3 | 1.06 (0.92, 1.22) | 0.00 | 0.88 |  |  |
| Age (years) |  |  |  |  | 0.79 |  |
| <60 | 11 | 1.04 (0.96, 1.12) | 0.00 | 0.80 |  |  |
| ≥60 | 3 | 1.04 (0.81, 1.34) | 66.30 | 0.05 |  |  |
| Gender |  |  |  |  | 0.23 |  |
| Male | 5 | 0.99 (0.86, 1.15) | 39.00 | 0.16 |  |  |
| Female | 6 | 1.10 (0.98, 1.24) | 0.00 | 0.64 |  |  |
| Follow-up (median, years) |  |  |  |  | 0.94 |  |
| ≤9.3 | 6 | 1.04 (0.93, 1.15) | 0.00 | 0.71 |  |  |
| >9.3 | 5 | 1.03 (0.95, 1.12) | 0.00 | 0.70 |  |  |
| Cancer location |  |  |  |  | 0.51 |  |
| Colon | 7 | 0.99 (0.90, 1.10) | 0.00 | 0.87 |  |  |
| Rectum | 7 | 1.05 (0.86, 1.29) | 48.80 | 0.07 |  |  |
| Quality scores |  |  |  |  | 0.40 |  |
| 7 | 2 | 1.00 (0.90, 1.10) | 0.00 | 0.77 |  |  |
| 8-9 | 9 | 1.06 (0.97, 1.15) | 0.00 | 0.83 |  |  |
| Study design |  |  |  |  | 0.84 |  |
| Prospective cohort | 9 | 1.03 (0.96, 1.11) | 0.00 | 0.77 |  |  |
| Case cohort | 2 | 1.01 (0.82, 1.25) | 0.00 | 0.67 |  |  |
| Multiple adjustments |  |  |  |  |  |  |
| Age |  |  |  |  | 0.44 |  |
| yes | 9 | 1.02 (0.96, 1.10) | 0.00 | 0.82 |  |  |
| no | 2 | 1.13 (0.89, 1.44) | 0.00 | 0.75 |  |  |
| BMI |  |  |  |  | 0.84 |  |
| yes | 9 | 1.03 (0.96, 1.11) | 0.00 | 0.77 |  |  |
| no | 2 | 1.01 (0.82, 1.25) | 0.00 | 0.67 |  |  |
| Family history |  |  |  |  | 0.69 |  |
| yes | 5 | 1.02 (0.94, 1.11) | 0.00 | 0.81 |  |  |
| no | 6 | 1.05 (0.95, 1.17) | 0.00 | 0.65 |  |  |
| smoking history |  |  |  |  | 0.85 |  |
| yes | 9 | 1.03 (0.96, 1.11) | 0.00 | 0.75 |  |  |
| no | 2 | 1.02 (0.87, 1.19) | 0.00 | 0.82 |  |  |
| alcohol intake |  |  |  |  | 0.79 |  |
| yes | 9 | 1.04 (0.96, 1.12) | 0.00 | 0.76 |  |  |
| no | 2 | 1.01 (0.87, 1.17) | 0.00 | 0.88 |  |  |
| ^1^P for heterogeneity within each subgroup with Q test. ^2^P for difference between subgroups with meta-regression analysis. | | | | | |  |
|  |  |  |  |  |  |  |

**Supplementary Table 5.** Subgroup analyses for biomarkers of Alpha-linolenic Acid in the top quartile compared with the bottom

| **Factors stratified** | **Biomarker** | | | | |  |
| --- | --- | --- | --- | --- | --- | --- |
|  | **N** | **RR(95% CI)** | **Heterogeneity** | | **P^2^** |  |
|  |  |  | **I^2^ (%)** | **P^1^** |  |  |
| Overall analysis | 5 | 0.85 (0.69, 0.96) | 0.00 | 0.83 |  |  |
| Tissue types (all biomarkers) |  |  |  |  | 0.86 |  |
| Adipose | 1 | 0.88 (0.47, 1.65) |  |  |  |  |
| Circulation | 4 | 0.83 (0.69, 0.99) | 0.00 | 0.65 |  |  |
| Tissue types (circulation) |  |  |  |  | 0.79 |  |
| Plasma | 2 | 0.85 (0.64, 1.14) | 0.00 | 0.36 |  |  |
| Erythrocyte | 2 | 0.81 (0.63, 1.03) | 0.00 | 0.40 |  |  |
| Age (years) |  |  |  |  | 0.87 |  |
| <60 | 4 | 0.82 (0.68, 0.99) | 0.00 | 0.65 |  |  |
| ≥60 | 2 | 0.88 (0.47, 1.65) | 86.00 | 0.01 |  |  |
| Gender |  |  |  |  | 0.50 |  |
| Male | 1 | 0.39 (0.16, 0.91) |  |  |  |  |
| Female | 2 | 1.13 (0.36, 3.69) | 79.80 | 0.03 |  |  |
| Cancer location |  |  |  |  | 0.34 |  |
| Colon | 2 | 0.62 (0.30, 1.25) | 79.60 | 0.03 |  |  |
| Rectum | 2 | 1.15 (0.59, 2.26) | 58.80 | 0.12 |  |  |
| ^1^P for heterogeneity within each subgroup with Q test. ^2^P for difference between subgroups with meta-regression analysis. | | | | | |  |
|  |  |  |  |  |  |  |
